# Sustained improvements by behavioural parent training for children with attention‐deficit/hyperactivity disorder: A meta‐analytic review of longer‐term child and parental outcomes

**DOI:** 10.1002/jcv2.12196

**Published:** 2023-09-04

**Authors:** Dominique P. A. Doffer, Tycho J. Dekkers, Rianne Hornstra, Saskia van der Oord, Marjolein Luman, Patty Leijten, Pieter J. Hoekstra, Barbara J. van den Hoofdakker, Annabeth P. Groenman

**Affiliations:** ^1^ Department of Child and Adolescent Psychiatry University of Groningen University Medical Center Groningen Groningen The Netherlands; ^2^ Accare Child Study Center Groningen The Netherlands; ^3^ Levvel, Academic Center for Child and Adolescent Psychiatry Amsterdam The Netherlands; ^4^ Department of Psychology University of Amsterdam Amsterdam The Netherlands; ^5^ Department of Child and Adolescent Psychiatry Amsterdam University Medical Centers (AUMC) Amsterdam The Netherlands; ^6^ KU Leuven Clinical Psychology Faculty of Psychology and Educational Sciences Leuven Belgium; ^7^ Department of Clinical, Neuro, and Developmental Psychology Vrije Universiteit Amsterdam Amsterdam Public Health Research Institute Amsterdam The Netherlands; ^8^ University of Amsterdam Research Institute of Child Development and Education Amsterdam The Netherlands; ^9^ Department of Clinical Psychology and Experimental Psychopathology University of Groningen Groningen The Netherlands

**Keywords:** attention‐deficit/hyperactivity disorder, behavioural parent training, longer‐term outcomes, meta‐analysis, parenting

## Abstract

**Background:**

Behavioural parent training is an evidence‐based intervention for children with attention‐deficit/hyperactivity disorder (ADHD), but little is known about the extent to which initial benefits are maintained.

**Aims:**

This meta‐analytic review investigated longer‐term (i.e., more than 2 months post‐intervention) child and parental outcomes of behavioural parent training for children with ADHD.

**Materials & Methods:**

We searched for randomized controlled trials and examined ADHD symptoms, behavioural problems, positive parenting, negative parenting, parenting sense of competence, parent‐child relationship quality, and parental mental health as outcomes. We included 27 studies (31 interventions; 217 effect sizes), used multilevel random‐effects meta‐analyses for between‐ and within‐group comparisons (pre‐intervention to follow‐up and post‐intervention to follow‐up), and explored twelve predictors of change.

**Results:**

Between pre‐intervention and follow‐up (*M* = 5.3 months), we found significant small‐to‐moderate between‐group effects of the intervention on ADHD symptoms, behavioural problems, positive parenting, parenting sense of competence and parent‐child relationship quality. Within‐group findings show sustained improvements in the intervention conditions for all outcome domains. There were few significant changes from post‐intervention to follow‐up. Additionally, the large majority of the individual effect sizes indicated sustained outcomes from post‐intervention to follow‐up. There were seven significant predictors of change in child outcomes, including stronger reductions in ADHD symptoms of girls and behaviour problems of younger children. In contrast with some meta‐analyses on short‐term effects, we found no differences between masked and unmasked outcomes on ADHD symptoms at follow‐up.

**Discussion & Conclusion:**

We conclude that behavioural parent training has longer‐term benefits for children's ADHD symptoms and behavioural problems, and for positive parenting behaviours, parenting sense of competence and quality of the parent‐child relationship.

## Graphical abstract

In this meta‐analytic review, we found that on the longer term (at least up to 5 months), behavioural parent training reduces children's ADHD symptoms and behavioural problems, and improves parents' skills, parenting sense of competence and the quality of the relationship between parents and their child.

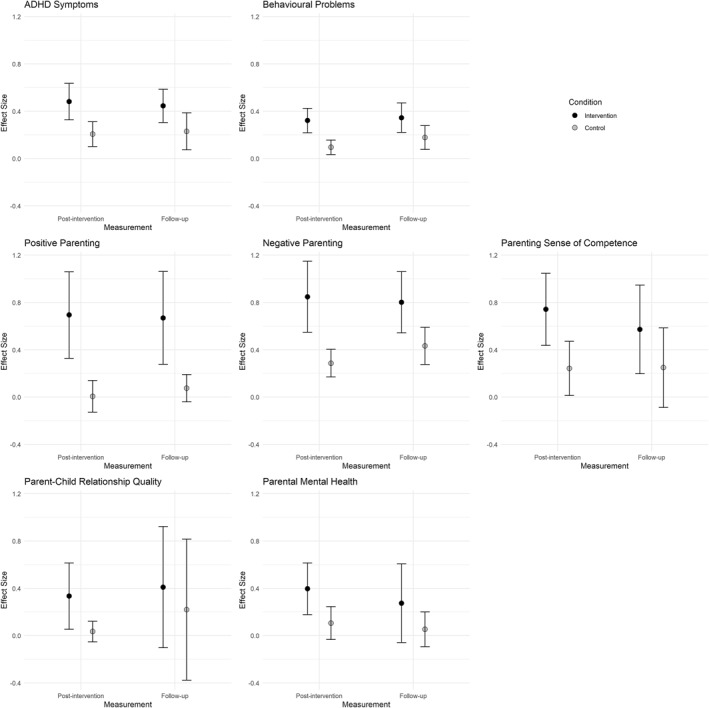

**Key points**

- Behavioural parent training is an evidence‐based intervention for children with ADHD, but little is known about the extent to which changes are maintained after the intervention has finished.
- A meta‐analysis of between‐ and within‐group effect sizes indicates sustained outcomes of behavioural parent training on children's ADHD symptoms and behavioural problems, and on positive parenting behaviours, parenting sense of competence and quality of the parent‐child relationship.
- Previous work reports smaller short‐term effects for ADHD symptoms on masked compared to unmasked outcome measures, however, we did not find such differences on longer‐term outcomes.
- Clinicians can be confident about the longer‐term outcomes of behavioural parent training for children with ADHD up to (on average) 5 months.
- A limitation of the literature is that it is often not described in detail which interventions families do or do not receive in the period between post‐intervention and follow‐up – which leaves room for several alternative explanations.

## INTRODUCTION

Behavioural parent training is an evidence‐based intervention for children with attention‐deficit/hyperactivity disorder (ADHD), as documented by multiple meta‐analyses and endorsed by practice guidelines (American Academy of Child and Adolescent Psychiatry, 2007; Daley et al., 2017; Dekkers et al., 2022; National Institute for Health and Care Excellence [NICE], 2018). The intervention has positive short‐term effects on children's ADHD symptoms and behavioural problems (Daley et al., 2014; Groenman et al., 2022; Hornstra et al., 2022; Lee et al., 2012; Rimestad et al., 2019). In addition, improvements in several short‐term parental outcomes are well documented, including more positive (e.g., positive reinforcement, monitoring) and less negative (e.g., harsh discipline, inconsistency) parenting, increased parenting sense of competence, higher quality of the parent‐child relationship, and improved parental mental health (Daley et al., 2014; Dekkers et al., 2022; Lee et al., 2012; Rimestad et al., 2019). However, the vast majority of trials and meta‐analyses, including our own (Dekkers et al., 2022; Hornstra et al., 2022), focused on short‐term effects. Therefore, little is known about the extent to which changes are maintained after the treatment has finished.

Two previous meta‐analyses investigated longer‐term outcomes (i.e., outcomes at least 2 months after the intervention ended) of behavioural parent training for children with ADHD. Rimestad et al. (2019) reported that improvements on (parent‐reported) ADHD symptoms and (both parent‐reported and observed) negative parenting sustained between immediate post‐intervention and follow‐up assessments. However, this meta‐analysis only included studies in preschoolers. Lee et al. (2012) similarly concluded that improvements for most child (i.e., increase in positive behaviour, decrease in disruptive behaviour) and parental outcomes (i.e., changes in child‐rearing behaviour) remained meaningful at follow‐up, although effect sizes were smaller than immediately post‐treatment. Since that meta‐analysis was performed more than 10 years ago, many studies on longer‐term outcomes have been published. In the present study, we built on these initial meta‐analyses by performing an updated meta‐analysis on longer‐term child and parental outcomes of behavioural parent training for ADHD from pre‐intervention to later follow‐up, including only longer‐term effects from follow‐up measurement(s) of randomized controlled trials in children of all age groups.

A second aim was to explore patterns of change (or lack thereof) from post‐intervention (i.e., immediate post‐intervention) to follow‐up (i.e., later follow‐up assessment(s)). Three patterns are possible: fade‐out effects (i.e., weakening of effects with time), sustained effects (i.e., no change between post‐intervention and follow‐up assessments), and sleeper effects (i.e., enlargement of effects with time). Fade‐out effects are theoretically plausible: continuously rewarding desired behaviour is crucial for children with ADHD given their marked instrumental learning difficulties, but this can be challenging to maintain over longer periods (Van der Oord & Tripp, 2020). Moreover, parents of children with ADHD often have increased ADHD symptoms themselves, potentially making it challenging to consistently apply the learned parenting techniques (Chronis‐Tuscano et al., 2011). In contrast, parents may also experience positive feedback loops when they experience that the newly learned parenting skills positively affect their child's behaviour, which makes it easier to maintain their newly learned skills in their daily life (i.e., sustained effects), or even leads to generalization of learned skills to other problem behaviours causing further decrease in problems (i.e., sleeper effects). In a meta‐analysis of parenting interventions for children with disruptive behaviour, changes on child disruptive behaviour on average sustained from post‐intervention to follow‐up, although 16% of the individual trials indicated fade‐out effects and 12% indicated sleeper effects (van Aar et al., 2017). Here, we adopted a similar approach to assess patterns of change of behavioural parent training for children with ADHD, by determining effect sizes between post‐intervention and follow‐up measurement(s) and looking at changes in child and parenting behaviour by calculating percentages of individual effect sizes that demonstrated fade‐out, sustained, and sleeper effects.

Because individual studies are inconsistent in the extent to which they suggest longer‐term effects, a third aim of our study was to explore under what conditions behavioural parent training is most effective on the longer term. We selected a broad range of participant, intervention, and study characteristics that may act as predictors,^1^ based on earlier (short‐term) meta‐analyses. Please note that these analyses should be regarded as hypothesis‐generating and not hypothesis‐confirming. We focused on the role of children's age, sex, presence of comorbid disruptive behaviour disorders, and initial immediate intervention success as participant characteristics; delivery (i.e., group, individual, or mixed), format (i.e., parent training or multimodal intervention) and duration as intervention characteristics; and type of control condition (i.e., active or waitlist), study quality, time lag between post‐measurement and follow‐up assessment(s), masking of outcome measures, rater type, and whether other treatments were allowed (for both conditions) after the post‐measurement, as study characteristics (Daley et al., 2014, 2017; Groenman et al., 2022; Hornstra et al., 2022; Lee et al., 2012; Rimestad et al., 2019; van Aar et al., 2017).

Generally, meta‐analyses looking at short‐term effects of interventions use between‐group effect sizes to estimate the effectiveness of the treatment of interest. Here, both between‐ and within‐group effect sizes were used to investigate our first two research aims. Although between‐group analyses are the gold standard when synthesizing outcomes of randomized controlled trials, the situation is much less straightforward when focusing on outcomes that occur after the immediate post‐measurement, when the controlled phase of the randomized controlled trial has ended. Then, typically, participants allocated to (waitlist) control conditions are offered the treatment of interest, making between‐group differences alone less informative, as these may be confounded by (other) treatments that were provided. Within‐group effect sizes therefore contain additional information when investigating the sustainability of treatment outcomes over time (Cuijpers et al., 2017), even though they do not account for other factors than the treatment itself that could explain improvements.

In sum, the overall aim of this meta‐analysis was to evaluate the longer‐term outcomes of behavioural parent training for children with ADHD. We examined (1) longer‐term between‐ and within‐group effects of behavioural parent training from pre‐intervention to follow‐up on child and parental outcomes; (2) between‐ and within‐group patterns of change from post‐intervention to follow‐up and (3) whether participant, intervention, and study characteristics were associated with longer‐term intervention effects from pre‐intervention to follow‐up, as well as from post‐intervention to follow‐up.

## MATERIALS AND METHODS

We used a similar methodology and updated the search from two earlier meta‐analyses on the short‐term effects of behavioural parent training for ADHD from our group (Dekkers et al., 2022; Hornstra et al., 2022). Parts of the methods may therefore overlap.

### Reporting guidelines

This meta‐analysis was registered at PROSPERO (record ID CRD42022344202) and Preferred Reporting Items for Systematic Reviews and Meta‐Analyses (PRISMA) guidelines were used (Moher et al., 2009; see Appendix S1 for the PRISMA checklist).

### Eligibility criteria

We included randomized controlled trials on behavioural parent training for children with ADHD. This was defined as an intervention that teaches parents techniques to manipulate the antecedents of the child's behaviour (stimulus control techniques, such as restructuring the environment) and/or techniques to manipulate behavioural contingencies (contingency management techniques, such as rewarding behaviour), to increase the occurrence of desired behaviours and decrease unwanted behaviours of their child (Evans et al., 2018). We only included randomized controlled trails, as these trials provide the highest level of evidence with the lowest risk of biases (Sterne et al., 2022).

Inclusion criteria were: (a) children met ADHD criteria according to DSM or ICD classifications (any edition), as measured by diagnostic interviews or questionnaires; (b) children were younger than 18 years; (c) parents in the intervention condition received behavioural parent training or a multimodal intervention with more than 50% of the intervention time spent with parents; (d) in the control condition parents received (I) an active non‐specific control treatment, (II) treatment as usual, or (III) no treatment (i.e., waitlist), and in case of multiple control conditions, the one with the highest quality was selected (I > II > III); (e) studies contained at least one follow‐up measurement that was conducted at least 2 months after post‐intervention; (f) studies measured at least one of the outcome measures as described below. Studies were excluded if (a) medication use was an entry criterion of the study; (b) medication was used as control condition; (c) studies primarily targeted children with comorbid ADHD (e.g., studies aimed at children with sleep disorders and ADHD).

### Outcome variables

Our primary outcome measures were children's ADHD symptoms and behavioural problems (i.e., symptoms of oppositional defiant disorder and/or conduct disorder) (Hornstra et al., 2022). As secondary outcome measures, we selected five parenting outcomes based on earlier meta‐analyses (Daley et al., 2014; Dekkers et al., 2022): positive parenting (e.g., display of positive reinforcement of non‐disruptive behaviour, monitoring, stimulation), negative parenting (e.g., display of punishment, harsh discipline, inconsistency), parenting sense of competence (e.g., self‐efficacy), quality of the parent‐child relationship (e.g., affection, sensitivity, responsiveness), and parental mental health (e.g., parental depression, parental ADHD).

### Search and screening process

The search described in Dekkers et al. (2022) and Hornstra et al. (2022) has been updated by including studies from May 13, 2020 up to June 10, 2022, using the following databases: PubMed, EMBASE + EMBASE CLASSIC, PsycINFO, CINAHL, ERIC, and Web of Science (see Appendix S2 for the PRISMA flowchart and Appendix S3 for the search terms per database). Potential studies were independently screened for inclusion using Rayyan (Ouzzani et al., 2016) by two out of four raters (TJD, RH, APG and DPAD). Also, for studies included in the earlier search (up to May 13, 2020), we searched for additional later reports of follow‐up outcomes in Google Scholar using the ‘cited by’ function. Disagreement was resolved by discussion.

### Data extraction

Information needed to calculate the standardized mean difference (SMD) and the standardized mean change score using change score standardization (SMCC; Morris & DeShon, 2002) was extracted for all outcome measures at the pre‐intervention, post‐intervention, and follow‐up measurement (there were no studies with multiple follow‐up measurements). In addition, we extracted (1) participant characteristics: children's age (i.e., sample mean age), sex (i.e., percentage male), comorbid disruptive behaviour disorders (i.e., percentage of children with disruptive behaviour disorders), and initial post‐intervention effects (i.e., information to calculate pretest‐posttest between‐group effect size); (2) intervention characteristics: delivery (i.e., group, individual, or mixed), format (i.e., parent training vs. multimodal intervention), and duration of the intervention (in minutes); (3) study characteristics: type of control condition (i.e., active (other treatment or treatment as usual) vs. waitlist control condition), study quality (i.e., percentage low risk of bias, and percentage high risk of bias), time lag between post‐intervention and follow‐up measurement (in months), masking of outcome measures (i.e., masked (teachers, clinicians and coders who were unaware of treatment condition) vs. unmasked outcome assessment), rater type (i.e., parent, teacher, clinician, or coder), and whether additional care was allowed after immediate post‐intervention measurement in the intervention and control group (i.e., yes or no). We contacted authors in case of missing information. Data extraction was performed independently by two out of four authors (TJD, RH, APG and DPAD). Again, disagreement was resolved by discussion.

### Data analytic approach

All analyses were performed in R (version 4.1.3; R Core Team, 2021) using the “metafor” (version 3.8–1; Viechtbauer, 2010) and “dmetar” packages (version 0.0.9000; Harrer et al., 2019). We used the SMD to calculate all between‐group effect sizes and the SMCC (with *r* = 0.50) for within‐group analyses (Morris & DeShon, 2002). The estimation of 0.50 is based on the correlation used in an earlier meta‐analysis on the effectiveness of parenting programs (Nowak & Heinrichs, 2008). We used multilevel random‐effects to assess the longer‐term effects of behavioural parent training, as all analyses contained three levels, that is, the sampling variance of the effect sizes (level 1), the variance between effect sizes from the same study (level 2), and the variance between effect sizes across studies (level 3) (Assink & Wibbelink, 2016). This approach accounts for the dependency between effect sizes within studies, for example, when two different measures were used to measure one outcome variable within one study.

To answer the first research question (i.e., effects from pre‐intervention to follow‐up), we pooled between‐group effect sizes (i.e., behavioural parent training vs. control condition) as well as within‐group effect sizes (i.e., only behavioural parent training), from pre‐intervention to follow‐up using random effects multilevel meta‐analysis. Additionally, sensitivity analyses were conducted using correlations of 0.30 and 0.70 (Borenstein, 2009; Rosenthal, 1993).

To answer the second research question (i.e., patterns of change after the intervention), we similarily pooled between‐ and within‐group effect sizes from post‐intervention to follow‐up. Moreover, we calculated percentages of sustained, fade‐out and sleeper effects for all outcome measures separately, by looking at the 95% confidence intervals of all between‐ and within‐group effect sizes from post‐intervention to follow‐up. Fade‐out effects were indicated if the 95% confidence interval was completely below zero, sustained effects if the 95% confidence interval included zero, and sleeper effects if the 95% confidence interval was completely above zero (Van Aar et al., 2017). Subsequently, we calculated the percentages of fade‐out, sustained and sleeper effects for all outcome measures.

For the third research question (i.e., predictive value of participant, intervention and study characteristics), we performed random‐effects multilevel meta‐regression analyses on the within‐group effect sizes (intervention condition) from pre‐intervention to follow‐up, as well as on the within‐group effect sizes (intervention condition) from post‐intervention to follow‐up (Konstantopoulos, 2011). We did not control for multiple testing, as the predictor analyses were hypothesis‐generating, rather than hypothesis‐confirming (Bender & Lange, 2001; Streiner & Norman, 2011).

To ensure that positive effect sizes represent beneficial effects of behavioural parent training, some effect sizes were recoded. Effect sizes of 0.2, 0.5 and 0.8 are interpreted as small, medium, and large effects respectively (Cohen, 1988). Continuous predictor analyses were only performed when at least 10 effect sizes were available, for categorical predictor analyses the threshold was at least four effect sizes per subgroup (Fu et al., 2010). In both cases, multiple effect sizes could be derived from one study.

For all analyses, we used *I* ^2^ statistics as an indication of heterogeneity, separated for between‐variables and between‐studies heterogeneity (levels 2 and 3, respectively). Publication bias was assessed using Egger's test for funnel plot assymetry on the pre‐intervention to follow‐up within‐group effect sizes (Egger et al., 1997). We performed trim‐and‐fill procedures to estimate the number of studies needed to counteract potential funnel plot assymetry (Duval & Tweedie, 2000). *P*‐curves were calcluated to estimate the evidential value of the findings and to rule out possible flexibility in the data analysis (Simonsohn et al., 2014).

### Risk of bias

The risk of bias of included studies was determined independently by two out of three authors (RH, APG and DPAD), using the Cochrane Collaboration's tool for assessing risk of bias in randomized trials (Higgins & Green, 2011). We assessed the following criteria: (1) random sequence generation; (2) allocation concealment; (3) blinding of outcome assessment; (4) incomplete outocome data; (5) selective reporting. In addition, vested interests were assessed.

## RESULTS

### Study selection

We included 27 studies on 31 interventions, together containing 217 relevant effect sizes (see Appendix S2 for the PRISMA flowchart). In total 1481 children were included in the intervention conditions, and 988 in the control conditions. The period between post‐measurement and follow‐up was 5.3 months on average (ranging from two to twelve months). See Table 1 for study characteristics.

**TABLE 1:** Study characteristics.

Study	Intervention (*n*)	Control (*n*)	Age	Sex (% boys)	Behaviour disorder (%)	Format/Delivery	Duration	Time lag follow‐up	Masked outcome	Rater
Abikoff et al. (2015)	HNC (63)	WL (34)	3.6	74	42	PT/I	480	6.8	Yes	P, T, Cl, Co
	NFPP (67)		3.6	74	42	PT/I	600	6.8	Yes	P, T, Cl, Co
Au et al. (2014)	Triple‐P (8)	WL (9)	7.7	94	N/A	PT/M	975	3.0	No	P
Chacko et al. (2009)	BPT (40)	WL (40)	7.9	71	71	MM/G	1350	3.0	No	P
	STEPP (40)		7.9	71	71	MM/G	1350	3.0	No	P
Chesterfield et al. (2021)	1‐2‐3‐Magic (28)	WL (40)	8.1	61	N/A	PT/G	360	6.0	No	P
Ferrin et al. (2014)	Psych. ed. (42)	AC (37)	10.7	80	30	PT/G	1080	12.0	No	P
Ferrin et al. (2020)	Psych. ed. (35)	TAU (34)	10.7	87	97	PT/G	720	6.0	No	P, T
Franke et al. (2020)	TPOL (27)	WL (26)	4.0	72	N/A	PT/I	480	6.0	No	P
Hoath & Sanders, (2002)	EGTP (9)	WL (11)	7.7	80	N/A	PT/G	574	3.0	No	P
Jiang et al. (2018)	CLAS (74)	TAU (51)	8.6	54	5	MM/M	1260	6.0	No	P
	PFT (74)		8.6	62	6	PT/M	1080	6.0	No	P
Lange et al. (2018)	NFPP (86)	TAU (75)	5.1	73	8	PT/I	600	8.3	Yes	P, T, Co
Larsen et al. (2021)	NFPP (86)	TAU (75)	5.1	73	8	PT/I	600	8.3	No	P
Matos et al. (2009)	PCIT (20)	WL (12)	5.0	N/A	98	PT/I	1305	3.5	No	P
Mautone et al. (2012)	FSS‐EE (24)	CARE (29)	N/A	72	30	MM/M	980	2.0	Yes	P, T, Co
Mikami et al. (2020)	PFC (84)	CARE (88)	8.7	74	27	PT/G	900	8.0	Yes	Co
Pfiffner et al. (2014)	CLAS (74)	TAU (51)	8.6	54	5	MM/M	1260	6.0	No	P, T
	PFT (74)		8.6	62	6	PT/M	1080	6.0	No	P, T
Pisterman et al. (1989)	BPT (23)	WL (23)	4.2	80	N/A	PT/M	720	3.0	Yes	Co
Pisterman et al. (1992b)	BPT (23)	WL (22)	4.1	84	N/A	PT/G	720	3.0	Yes	Co
Pisterman et al. (1992a)	BPT (46)	WL (45)	4.2	82	N/A	PT/M	720	3.0	No	P
Power et al. (2012)	FSS (92)	CARE (96)	N/A	68	27	MM/M	980	3.0	No	P, T
Sibley et al. (2016)	STAND (67)	TAU (61)	12.7	65	58	MM/I	740	6.0	No	P, T, Ch
Sibley et al. (2021)	STAND (138)	TAU (140)	14.0	70	51	MM/I	600	9.8	Yes	P, T, Ch, S
Smit et al. (2022)	PFC (84)	CARE (88)	8.7	74	27	PT/G	900	8.0	Yes	P, Co
Sonuga‐Barke et al. (2001)	PT (30)	PC&S (28)	3.5	62	N/A	PT/I	480	3.4	Yes	P, Cl, Co
Sonuga‐Barke et al. (2004)	PT (59)	WL (30)	3.5	N/A	N/A	PT/I	480	3.4	Yes	P, Cl
Sonuga‐Barke et al. (2018)	IY (131)	TAU (42)	3.5	71	73	PT/G	1620	6.0	Yes	P, T, Co
	NFPP (133)		3.6	75	74	PT/I	1080	6.0	Yes	P, T, Co
Thompson et al. (2009)	NFPP (17)	TAU (13)	4.5	76	N/A	PT/I	600	2.0	Yes	P, Cl, Co
Van den Hoofdakker et al. (2007)	BPT (47)	RCC (47)	7.4	76	76	PT/I	1440	6.0	No	P

Abbreviation: N/A, not available.

Intervention: BPT, Behavioural Parent Training; CLAS, Child Life and Attention Skills; EGTP, Enhanced Group Triple P; FSS, Family School Success; FSS‐EE, Family School Success Early Elementary; HNC, Helping the Noncompliant Child; IY, Incredible Years; NFPP, New Forest Parenting Programme; PCIT, Parent–Child Interaction Therapy; PFC, Parental Friendship Coaching; PFT, Parent‐Focused Treatment; Psych. ed.,Psychoeducation; STAND, Supporting Teens' Academic Needs Daily; STEPP, Strategies to Enhance Positive Parenting; TPOL, Triple P Online.

Control: AC, active control; CARE, Coping With ADHD Through Relationships and Education; PC&S, Parent Counselling and Support; RCC, routine clinical care; TAU, treatment as usual; WL, waitlist.

Age: mean age of the children in the intervention condition in years.

Sex: percentage of boys in intervention condition.

Behaviour disorder: percentage of children with comorbid disruptive behaviour disorders.

Format: MM, multimodal; PT, parent training.

Delivery: G, group; I, individual; M, mixed.

Duration: total duration of intervention in minutes.

Time lag follow‐up: time lag from post‐intervention to follow‐up in months.

Masked outcome: whether a masked outcome was included as outcome measure (yes or no).

Rater: Ch, child; Cl, clinician; Co, coder; P, parent; S, school; T, teacher.

### Risk of bias

Risk‐of‐bias analyses are available in Appendix S4. Interrater reliability of risk of bias scoring was high (*κ* = 0.91). Half of the studies included masked measures and most studies reported complete outcome data. Generally, reported information was insufficient to determine risk of bias for the categories random sequence generation, allocation concealment, selective outcome reporting, and vested interests.

### Main effects (pre‐intervention to follow‐up)

**FIGURE 1:**
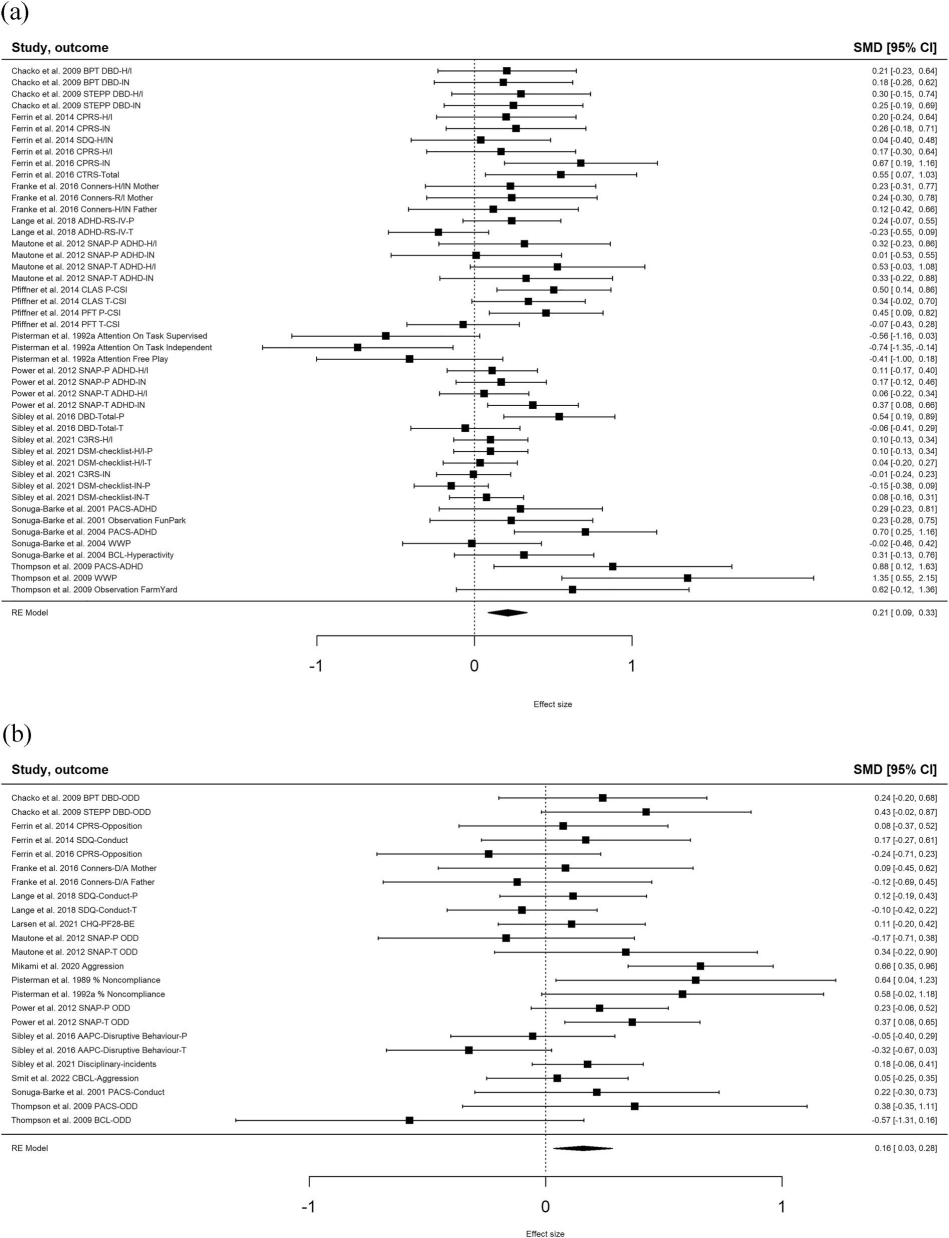
Forest plot of between‐group effect sizes from pre‐intervention to follow‐up measurement on all outcome measures for both primary outcomes. (A) ADHD symptoms. BCL, Behaviour Check List; BPT, Behavioural Parent Training; C3RS, Conners‐3 Parent Short Form Rating Scale; CLAS, Child Life and Attention Skills; CPRS, Conners Parent Rating Scale; CSI, Child Symptom Inventory; CTRS, Conners Teacher Rating Scale; DBD, Disruptive Behaviours Disorders rating scale; DSM, Diagnostic and Statistical Manual of Mental Disorders; H, hyperactivity; I, impulsivity; IN , inattention; IV, 4^th^ edition; P, parent; PACS, Parental Account of Childhood Symptoms; PFT, Parent‐Focused Treatment; R, restlessness; RS, rating scale; SNAP, Swanson, Nolan, and Pelham Questionnaire; STEPP, Strategies to Enhance Positive Parenting; T, teacher; WWP, Werry‐Weiss‐Peters‐Hyperactivity scale. (B) Behavioural problems. A, aggression; AAPC, Adolescent Academic Problems Checklist; BCL, Behaviour Check List; BPT, Behavioural Parent Training; CBCL, Child Behaviour Checklist; CHQ‐PF28‐BE, Child Health Questionnaire Parent Form 28 – Behaviour subscale; CPRS, Conners Parent Rating Scale; D, defiance; DBD, Disruptive Behaviours Disorders rating scale; ODD,oppositional defiant disorder; P, parent; PACS, Parental Account of Childhood Symptoms; SDQ, Strengths and Difficulties Questionnaire; SNAP, Swanson, Nolan, and Pelham Questionnaire; STEPP, Strategies to Enhance Positive Parenting; T, teacher.

**FIGURE 2:**
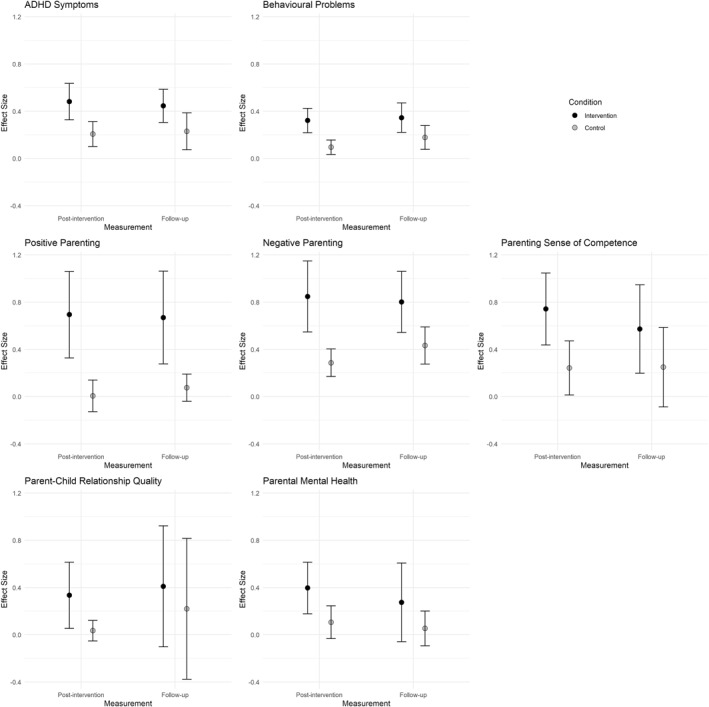
Within‐group effect sizes of the intervention and control condition for all outcomes. These figures are for visual purposes only and illustrate within‐group effect sizes from pre‐intervention to post‐intervention at ‘Post‐intervention’, and from pre‐intervention to follow‐up at ‘Follow‐up’, for the intervention and control condition separately. For test statistics, see Table 2. Only those studies that followed up the intervention condition as well as the control condition were included in these figures, therefore the effect sizes displayed may be slightly different than the effect sizes in Table 2.

Between‐group effect sizes (see Figure 1 for forest plots) indicated significant differences between behavioural parent training and control conditions from pre‐intervention to follow‐up favouring parent training, in ADHD symptoms (*SMD* = 0.21, 95% CI [0.09, 0.33]), behavioural problems (*SMD* = 0.16, 95% CI [0.03, 0.28]), positive parenting (*SMD* = 0.60, 95% CI [0.19, 1.00]), parenting sense of competence (*SMD* = 0.38, 95% CI [0.13, 0.62]), and the parent‐child relationship quality (*SMD* = 0.21, 95% CI [0.01, 0.41]), but not in negative parenting (*SMD* = 0.32, 95% CI [‐0.07, 0.72]), and parental mental health (*SMD* = 0.24, 95% CI [‐0.04, 0.52]). In addition, we found significant small‐to‐large within‐group effect sizes of behavioural parent training from pre‐intervention to follow‐up on all outcome domains (see Appendix S5 for forest plots). Sensitivity analyses showed that the within‐group findings were highly similar when we used other values for the correlation between measurements (see Appendix S6). Between‐ and within‐group effect sizes of the intervention condition are displayed in Table 2 and within‐group effect sizes of the control condition in Appendix S7. In addition, Figure 2 is a visualization of within‐group effect sizes of both conditions. In general, we found substantial heterogeneity between effect sizes (see Table 2 for detailed information).

**TABLE 2:** Between‐ and within‐group (intervention condition) effects per outcome domain.

		*k*	*n‐es*	ES	95% CI	*I* ^2^ (level 2, %)	*I* ^2^ (level 3, %)
ADHD symptoms	Between pre – follow‐up	16^a^	46^a^	0.21**	0.09–0.33	4.2	50.6
	Between post – follow‐up	15	43	−0.08	−0.18 to 0.02	0.0	32.6
	Between pre – post	15	43	0.29**	0.13–0.46	1.1	68.9
	Within pre – follow‐up	23^a^	72^a^	0.53***	0.40–0.66	51.6	36.7
	Within post – follow‐up	22	69	0.06	−0.00 to 0.12	46.2	15.9
	Within pre – post	22	69	0.53***	0.39–0.67	54.8	34.6
Behavioural problems	Between pre – follow‐up	17	24	0.16*	0.03–0.28	0.0	42.3
	Between post – follow‐up	17	24	−0.05	−0.20 to 0.09	0.0	55.4
	Between pre – post	17	24	0.19***	0.10–0.27	0.0	0.0
	Within pre – follow‐up	26	52	0.47***	0.33–0.61	6.1	79.0
	Within post – follow‐up	26	52	0.02	−0.06 to 0.09	49.4	15.4
	Within pre – post	26	52	0.47***	0.34–0.61	28.2	57.3
Positive parenting	Between pre – follow‐up	9	16	0.60**	0.19–1.00	50.1	36.7
	Between post – follow‐up	9	16	−0.14	−0.33 to 0.05	0.0	43.0
	Between pre – post	9	16	0.79***	0.46–1.11	84.0	0.0
	Within pre – follow‐up	14	21	0.66***	0.38–0.95	91.2	2.6
	Within post – follow‐up	14	21	−0.06	−0.15 to 0.02	0.0	33.1
	Within pre – post	14	21	0.70***	0.43–0.98	93.1	0.0
Negative parenting	Between pre – follow‐up	8	17	0.32	−0.07 to 0.72	55.4	29.8
	Between post – follow‐up	8	17	−0.16*	−0.30 to −0.03	0.0	11.4
	Between pre – post	8	17	0.49*	0.08–0.91	52.8	33.5
	Within pre – follow‐up	9	20	0.79***	0.57–1.02	73.8	5.3
	Within post – follow‐up	9	20	0.01	−0.07 to 0.09	0.0	0.0
	Within pre – post	9	20	0.79***	0.51–1.06	57.2	25.6
Parenting sense of competence	Between pre – follow‐up	9^a^	16^a^	0.38**	0.13–0.62	9.6	60.6
	Between post – follow‐up	8	14	−0.13*	−0.24 to −0.01	4.3	0.0
	Between pre – post	8	14	0.50***	0.26–0.74	0.0	63.3
	Within pre – follow‐up	13^a^	21^a^	0.54***	0.27–0.82	0.0	89.2
	Within post – follow‐up	12	19	−0.03	−0.10 to 0.04	0.0	0.0
	Within pre – post	12	19	0.68***	0.45–0.90	0.0	81.4
Parent‐child relationship quality	Between pre – follow‐up	8	11	0.21*	0.01–0.41	32.4	19.9
	Between post – follow‐up	8	11	−0.04	−0.22 to 0.14	2.7	37.0
	Between pre – post	8	11	0.27*	0.05–0.49	5.5	50.5
	Within pre – follow‐up	10	13	0.34*	0.00–0.68	44.0	49.0
	Within post – follow‐up	10	13	−0.02	−0.35 to 0.31	93.8	0.0
	Within pre – post	10	13	0.38**	0.13–0.62	88.0	0.0
Parental mental health	Between pre – follow‐up	9^a^	13^a^	0.24	−0.04 to 0.52	35.1	34.5
	Between post – follow‐up	8	12	−0.01	−0.15 to 0.13	0.0	0.0
	Between pre – post	8	12	0.26*	0.05–0.47	26.4	18.3
	Within pre – follow‐up	14^a^	18^a^	0.39**	0.16–0.63	0.0	86.5
	Within post – follow‐up	13	17	−0.01	−0.11 to 0.09	0.0	37.2
	Within pre – post	13	17	0.45***	0.29–0.60	40.9	28.4

*Note*: Effect sizes (ES) represent standardized mean differences (between‐group) and standardized mean change scores using change score standardization (within‐group), with 95% confidence intervals. Positive effect sizes indicate beneficial effects of the intervention for all outcomes. *I* ^2^ represents between‐variables and between‐studies heterogeneity (level 2 and 3 respectively). Within effect sizes represent effect sizes within the intervention condition.

Abbreviations: CI, confidence interval; *k*, number of studies; *n‐es*, number of effect sizes.

^a^ Sonuga‐Barke et al. (2004) only reported pre‐measurement and later follow‐up, and therefore the number of studies and effect sizes differs from the other comparisons.

***p < 0.001, **p < 0.01, *p < 0.05.

### Patterns of change (post‐intervention – follow‐up)

Generally, results indicate that initial changes sustained in the period between the end of the intervention and the follow‐up measurement. Most between‐ and within‐group effect sizes of the difference between post‐intervention and follow‐up measurements were not significant (see Appendix S5 for forest plots), except for the between‐group effect sizes of negative parenting (*SMD* = −0.16, 95% CI [−0.30, −0.03]) and parenting sense of competence (*SMD* = −0.12, 95% CI [−0.24, −0.01]), where group differences decreased from post‐intervention to follow‐up. Moreover, both between‐ and within‐group analyses indicated that the large majority of the individual effect sizes sustained (Table 3). Only 7% of the within‐group effect sizes demonstrated fade‐out effects and 11% sleeper effects, while for between‐group analyses we found 10% fade‐out effects and 3% sleeper effects. There were no obvious factors that might have been related to the large number of within‐group sleeper effects on ADHD symptoms. However, we observed that outcomes rated by teachers were disproportionately often classified as sleeper effects (i.e., 50% of all sleeper effects were teacher‐rated). Additionally, we performed a sensitivity analysis, in which we found no difference between pre‐intervention to post‐intervention versus pre‐intervention to follow‐up effect sizes, thus indicating sustained outcomes as well (see Appendix S8 for detailed results).

**TABLE 3:** Numbers and percentages of fade‐out, sustained and sleeper effects from post‐intervention to follow‐up.

	Within‐group effect sizes						Between‐group effect sizes					
	Fade‐out effects		Sustained effects		Sleeper effects		Fade‐out effects		Sustained effects		Sleeper effects	
	*k*	*n*‐es (%)	*k*	*n*‐es (%)	*k*	*n*‐es (%)	*k*	*n*‐es (%)	*k*	*n*‐es (%)	*k*	*n*‐es (%)
ADHD symptoms	1	1 (1.4)	22	50 (72.4)	7	18 (26.1)	2	2 (4.7)	15	39 (90.7)	1	2 (4.7)
Behavioural problems	2	2 (3.8)	24	46 (88.5)	4	4 (7.7)	1	2 (8.3)	15	21 (87.5)	1	1 (4.2)
Positive parenting	2	3 (14.3)	11	17 (81.0)	1	1 (4.8)	2	3 (18.8)	8	13 (81.3)	0	0 (0.0)
Negative parenting	1	1 (5.0)	9	19 (95.0)	0	0 (0.0)	2	2 (11.8)	7	15 (88.2)	0	0 (0.0)
Parenting sense of competence	1	1 (5.3)	12	18 (94.7)	0	0 (0.0)	1	1 (7.1)	8	13 (92.9)	0	0 (0.0)
Parent‐child relationship quality	3	4 (30.8)	8	8 (61.5)	1	1 (7.7)	1	1 (9.1)	7	9 (81.8)	1	1 (9.1)
Parental mental health	2	2 (11.8)	11	15 (88.2)	0	0 (0.0)	1	1 (8.3)	7	11 (91.7)	0	0 (0.0)

*Note*: *k*, number of studies, *n‐es*, number of effect sizes.

### Robustness analyses

Funnel plot asymmetry was indicated by Egger's test on behavioural problems (*t* = 2.99, *p* < 0.01), positive parenting (*t* = 3.03, *p* < 0.01), and negative parenting (*t* = 2.44, *p* = 0.03), potentially indicating publication bias. On outcome domains ADHD symptoms (*t* = 1.07, *p* = 0.29), parenting sense of competence (*t* = 0.89, *p* = 0.39), parent‐child relationship quality (*t* = 1.82, *p* = 0.10), and parental mental health (*t* = 0.42, *p* = 0.68), there were no indications of publication bias. Trim‐and‐fill analyses (Duval & Tweedie, 2000) indicated that five studies were missing on the left side of the funnel plot for ADHD symptoms and positive parenting, six for behavioural problems and negative parenting, one for parenting sense of competence, and three for parental mental health. Corrected effect sizes taking these potentially missing studies into account were somewhat lower, but still significant. Funnel plots on all outcome domains, and trim‐and‐fill analyses with corrected effect sizes where applicable are presented in Appendix S9 and S10.

P‐curves (Simonsohn et al., 2014) for all outcome domains were right‐skewed and indicated evidential value of findings (i.e., all tests for right skewness: *p* < 0.01). Only the power of the *p*‐curve for the outcome domain parent‐child relationship quality was below 80% (i.e., 70%), which was logical as the *p*‐curve was only based on 4 significant *p*‐values; for all other outcome domains power was high (i.e., 82% or higher). There were no signs of flexibility in data‐analysis. *P*‐curves can be found in Appendix S11, Figures S5.1–S5.7.

Leave‐one‐out analyses were performed to determine whether effect sizes were dependent on single studies. On most outcome domains, the influence of single studies was minimal, but for positive and negative parenting the effect sizes decreased substantially after leaving out two particular studies (Pisterman et al., 1989, 1992b; see Appendix S12 for an overview of the range and the minimum and maximum effect size on all outcome measures). Additionally, sensitivity analyses indicated that our results were independent of the discontinuation rates in studies (see Appendix S13 for detailed results).

Overall, although some analyses indicate publication bias on some outcome domains, with most pronounced effects on positive and negative parenting, the main effects appear robust based on trim‐and‐fill analyses, *p*‐curves and leave‐one‐out analyses.

### Meta‐regression analyses on possible characteristics associated with change

We investigated whether participant, intervention, and study characteristics were related to the within‐group effect sizes on both primary outcome measures from pre‐intervention to follow‐up and from post‐intervention to follow‐up. Detailed results of these analyses are presented in the Appendix S14, Tables S7.1–S7.6. Please note that all meta‐regression analyses should be regarded as hypothesis‐generating and not hypothesis‐confirming.

#### Participant characteristics

Sex of the child was associated with effect sizes from pre‐intervention to follow‐up on ADHD symptoms, but not on behavioural problems, with slightly lower effects for boys than girls. Age was related to effect sizes from pre‐intervention to follow‐up on behavioural problems, but not on ADHD symptoms, with larger effect sizes in younger children. The initial success of the intervention (as measured on both child outcome domains) was associated with effect sizes from pre‐intervention to follow‐up on ADHD symptoms and behavioural problems. Furthermore, initial intervention success on parental mental health was associated with more positive longer‐term child outcomes. Comorbidity with disruptive behaviour disorders was not a significant predictor. None of the participant characteristics were associated with post‐intervention to follow‐up effect sizes.

#### Intervention characteristics

The format of the intervention was associated with effect sizes from pre‐intervention to follow‐up on behavioural problems, but not on ADHD symptoms, with larger effect sizes for interventions consisting of parent training only. A mixed delivery method (both individual and group sessions) was related to larger effect sizes compared to a group delivery method on ADHD symptoms, but not on behavioural problems, from pre‐intervention to follow‐up. Intervention duration was not associated with effects sizes from pre‐intervention to follow‐up, and none of the intervention characteristics were related to effect sizes from post‐intervention to follow‐up.

#### Study characteristics

The type of control condition was related to effect sizes from pre‐intervention to follow‐up on behavioural problems, but not on ADHD symptoms, with larger effect sizes for interventions with passive relative to active control conditions. Study quality and differences between studies in the time lag from post‐intervention to follow‐up were not associated with effect sizes at follow‐up.

Effect sizes from pre‐intervention to follow‐up were similar for masked and unmasked raters on ADHD symptoms and behavioural problems. From post‐intervention to follow‐up, effect sizes were significantly larger for masked relative to unmasked raters, which we found on both ADHD symptoms and behavioural problems. Because effect sizes based on masked and unmasked measures in some cases came from different studies, these findings may be confounded with other study characteristics. Therefore, we conducted sensitivity analyses including only those studies that included both masked and unmasked measures of either ADHD symptoms (six studies) or behavioural problems (four studies). The results were similar for ADHD symptoms, with significantly larger effect sizes for masked relative to unmasked raters. For behavioural problems, the difference between masked and unmasked outcomes was no longer significant. For details on this sensitivity analysis, see Appendix S15.

Rater type was related to effect sizes from pre‐intervention to follow‐up on ADHD symptoms, with largest effect sizes for clinicians and parents, significantly smaller but still significant effect sizes for teachers, and an effect size around zero for coders. Moreover, teacher‐rated outcomes were associated with larger effect sizes compared to other raters from post‐intervention to follow‐up. For behavioural problems, we could only compare parent‐ and teacher‐rated outcomes, as there were insufficient studies including coders or clinicians as raters. Effect sizes from pre‐intervention to follow‐up were similar for both raters, but again, teacher‐rated outcomes predicted larger effect sizes from post‐intervention to follow‐up.

Too little information was reported in the studies to conduct any analyses on whether (other) treatment after the post‐measurement was allowed for children in the control and intervention conditions.

## DISCUSSION

The primary aim of this meta‐analysis was to investigate the longer‐term outcomes of behavioural parent training for children with ADHD. On both primary outcome domains (i.e., children's ADHD symptoms and behavioural problems), small significant between‐group effect sizes were found from pre‐intervention to follow‐up. Between‐group effect sizes indicated significant improvements on some secondary outcome measures as well (i.e., positive parenting, parenting sense of competence and parent‐child relationship quality), but not on negative parenting and parental mental health. Our within‐group analyses indicated sustained effects even more convincingly, as all within‐group effect sizes from pre‐intervention to follow‐up were significant.

As a secondary aim, we assessed post‐intervention patterns of change. The large majority of the individual effect sizes from post‐intervention to follow‐up indicated sustained outcomes (Table 3). These findings are in line with earlier findings, where parenting interventions for disruptive child behaviours lead to sustained reductions of disruptive behaviours (van Aar et al., 2017). Percentages of fade‐out and sleeper effects were somewhat higher in that study (i.e., 16% and 12% respectively). A difference between the current study and the one from Van Aar et al. (2017) worth mentioning is that their included studies contained a follow‐up period ranging from 1 month to 3 years, where sometimes multiple follow‐up measurements were conducted, while the mean follow‐up period in our meta‐analysis ranged from 2 months to 1 year, and none of the studies reported multiple follow‐up measurements. Possibly, more fade‐out and/or sleeper effects would occur during a longer follow‐up period. However, both our study and Van Aar et al. (2017) did not find a moderating effect of the length of the follow‐up period. Together, our findings indicate sustained effects of behavioural parent training after the intervention has ended, yet studies with longer follow‐up periods are needed.

Also on an aggregated level, both between‐ and within‐group outcomes indicate sustained improvements for most outcome measures, however for a few secondary outcome measures, between‐ and within‐group outcomes differed. We found no significant between‐group effect from pre‐intervention to follow‐up for negative parenting and parental mental health, and a small significant negative effect from post‐intervention to follow‐up for negative parenting and parenting sense of competence. Within‐group analyses allowed us to further interpret these between‐group findings. We found significant improvements in the control conditions for negative parenting and parenting sense of competence from post‐intervention to follow‐up, while improvement in the intervention conditions on these outcomes seemed to sustain (also see Appendix S7, Table S2.1). Consequently, this led to smaller (or even negative) between‐group effect sizes.

The mechanisms behind the improvements in the control condition are unknown. One explanation could be that families assigned to waitlist control conditions are somehow motivated to not demonstrate improvements during the study (i.e., nocebo effect), to ultimately receive the initially desired treatment (Furukawa et al., 2014). Additionally, a second explanation could be that families assigned to more passive control conditions received (other) interventions after the post‐intervention assessment, and longer‐term improvements seen in our analyses therefore indicate effects of these interventions. The same alternative explanation could hold for the intervention condition, such that the changes that we found at follow‐up could also be explained by families seeking alternative treatment after receiving behavioural parent training. However, families in the control condition are potentially more likely to seek treatment after post‐measurement, as they did not receive the treatment of interest. We planned to test whether any treatments received by families after post‐measurement predicted longer‐term outcomes, but unfortunately only 25% of the studies reported some information on received treatments between post‐intervention and follow‐up. These numbers did not allow for meaningful analyses. This is an important limitation of the literature, as it is currently unclear whether findings are confounded by (other) interventions that families received.

Although the main findings were consistent, substantial heterogeneity was observed between effect sizes. Our explorative analyses of potential associated characteristics revealed a small effect for children's sex (i.e., more improvements in ADHD symptoms in girls than boys), and age (i.e., more improvements in behavioural problems in younger children). Similar patterns have been found in earlier meta‐analyses of immediate effects (Daley et al., 2014; Dekkers et al., 2022). In addition, findings indicate that larger immediate changes in ADHD symptoms, behavioural problems, and parental mental health may be related to better longer‐term child outcomes. Parental mental health was already proposed to be relevant for intervention success (Chacko et al., 2015). Moreover, providing only parent training was found to be more effective in decreasing behavioural problems than multimodal interventions, a finding which was similar to our findings on short‐term outcomes (Dekkers et al., 2022). However, we did not find this difference for ADHD symptoms. An explanation might be that 80% of the multimodal interventions added a school component and therefore focused, compared to parent training only interventions, more on improving academic functioning, such as time management and planning in school (Mautone et al., 2012; Power et al., 2012; Sibley et al., 2016, 2021). The focus on academic functioning might make it more likely to achieve improvements in ADHD symptoms rather than behavioural problems. Furthermore, a mixed delivery method (both individual and group sessions) is possibly related to higher longer‐term improvements in ADHD symptoms, compared to group‐based programs. While individual sessions provide the opportunity to tailor the treatment to individual needs, group sessions can facilitate the sharing of experiences and social support between parents (Daley et al., 2017). No associations were found with other participant (i.e., comorbid disruptive behaviour disorders) or intervention (i.e., duration) characteristics. Regarding study characteristics, we found that studies with a passive instead of an active control condition had better outcomes (for behavioural problems, not ADHD symptoms).

Though masked and unmasked effect sizes did not differ from pre‐intervention to follow‐up, masked raters reported significantly more improvements compared to unmasked raters from post‐intervention to follow‐up, on both primary outcome measures. Including only those studies that included both masked and unmasked outcomes, results remained significant for ADHD symptoms, but turned insignificant for behavioural problems. These findings suggest that unmasked raters may be best able to observe improvements in ADHD symptoms immediately after the intervention has ended, while it takes longer for masked raters to observe these improvements, resulting in different ratings from pre‐intervention to post‐intervention (Daley et al., 2014; Sonuga‐Barke et al., 2013), that converge at the follow‐up measurement. Our finding that 50% of the sleeper effects on our primary outcome measures were teacher‐reported outcomes corroborates with this pattern. Possibly, generalization of parent training improvements to other contexts than the home setting (e.g., school settings) primarily occurs at a later stage.

Results also indicated that rater type might be associated with effect sizes from pre‐intervention to follow‐up on ADHD symptoms, in which parents and clinicians were most likely to report change in ADHD symptoms (compared to teachers and coders), suggesting that these raters were most sensitive in reporting treatment effects. This is not surprising given that treatments were mainly clinic‐based and focused on parents. However, it could also indicate some form of expectation bias (Williams et al., 2012). Teachers reported improvements as well, but effect sizes were somewhat lower compared to parents and clinicians. Coders did not observe change in ADHD symptoms in play situations (i.e., the only observation outcome in the included studies). Our results on different rater types are in line with the methodological concerns in measuring outcomes mentioned by Sibley et al. (2023). Moreover, the findings also align with research showing that parent‐ or teacher‐reported behaviour often does not correlate with more objective measures like observations or tasks (Staff et al., 2021; Toplak et al., 2013). This could indicate that grouping outcomes of raters unaware of treatment condition (e.g., teachers in case of behavioural parent training) and observations as masked assessments is not straightforward, as effects of parent training reported by teachers (often classified as masked) (Daley et al., 2014; Sonuga‐Barke et al., 2013) can differ substantially from effects on observation measures in terms of magnitude.

Although our study has several strong points including detailed analyses of between‐ and within‐group findings, inclusion of a large number of studies and effect sizes, and the use of state‐of‐the‐art multilevel meta‐analytic procedures, a few limitations warrant mentioning. First, the use of within‐group effect sizes in meta‐analyses is criticized by some, for example, because of the way in which correlations between pre‐post scores are calculated and accounted for (Cuijpers et al., 2017) or because effects may be inflated due to factors not related to the intervention. The use of fixed values for pre‐post correlations could have impacted our estimations of the effect sizes, even though our sensitivity analyses show similar results at different correlation values (also see Appendix S6). However, Cuijpers and colleagues also mention within‐group effect sizes can be useful, for example, during follow‐up in trials when no control group is available anymore. Especially in situations where randomization is not in place after an initial randomization period, within‐group effect sizes can be helpful in interpretating results of between‐group effect sizes. A second limitation is that most studies had relatively brief follow‐up periods (on average 5.3 months), and therefore we were unable to determine changes beyond this term. Future studies with much longer follow‐up periods are needed to gain more insight in long‐term treatment effects. An encouraging finding, however, was that length of the follow‐up period did not predict effects, which indicates that changes after 2 months were likely similar to effects after 12 months. Third, our meta‐regression analyses should be regarded as hypothesis‐generating, rather than hypothesis‐confirming. Correcting for multiple testing is not advised for this approach (Bender & Lange, 2001; Streiner & Norman, 2011). However, our results on associated characteristics should be interpreted with caution and have to be confirmed, or falsified, by future studies.

In sum, both between‐ and within‐group effect sizes indicate similar results, in which improvements on both primary outcome measures and most secondary outcome measures sustain. This consistent pattern of sustained improvements of behavioural parent training implies that clinicians can be relatively confident about the longer‐term (i.e., at least up to approximately 5 months) outcomes of behavioural parent training, which is important when discussing different treatment options with parents.

## CONCLUSION

In this meta‐analytic review, we found that on the longer term (i.e., at least up to 5 months), behavioural parent training reduces children's ADHD symptoms and behavioural problems, and improves parents' skills, parenting sense of competence and the quality of the relationship between parents and their child. Further randomized controlled trials with longer follow‐up periods (i.e., more than 1 year) are required to draw stronger conclusions about the longer‐term effects of behavioural parent training for children with ADHD.

## AUTHOR CONTRIBUTIONS

**Dominique P. A. Doffer**: Data curation; formal analysis; investigation; project administration; visualization; writing – original draft; writing – review & editing. **Tycho J. Dekkers**: Conceptualization; data curation; funding acquisition; investigation; supervision; writing – original draft; writing – review & editing. **Rianne Hornstra**: Data curation; project administration; writing – review & editing. **Saskia van der Oord**: Funding acquisition; supervision; writing – review & editing. **Marjolein Luman**: Funding acquisition; supervision; writing – review & editing. **Patty Leijten**: Funding acquisition; supervision; writing – review & editing. **Pieter J. Hoekstra**: Funding acquisition; supervision; writing – review & editing. **Barbara J. van den Hoofdakker**: Conceptualization; funding acquisition; investigation; supervision; writing – review & editing. **Annabeth P. Groenman**: Conceptualization; data curation; formal analysis; funding acquisition; methodology; supervision; writing – review & editing.

## CONFLICT OF INTEREST STATEMENT

Prof. Dr. van der Oord has co‐developed a planning‐focused and solution‐focused treatment and other behavioural treatments, but has no financial interest in any of these. She has received research grants from ZonMw and the Research Foundation Flanders (FWO). She was an advisor of the Dutch ADHD guideline groups and is a member of a working group on ADHD of the Superior Health Council of Belgium. Dr. Luman has co‐developed a self‐help teacher training program, without financial interests. She has received research grants from ZonMw and was an advisor of the Dutch ADHD guideline groups. Prof. Dr. van den Hoofdakker has received royalties as one of the editors of “Sociaal Onhandig” (published by Van Gorcum), a Dutch book for parents that can be used in parent training. She has been involved in the development and evaluation of several parent and teacher training programs, without financial interests. She has been a member of Dutch ADHD guideline and practice standard groups. Dr. Dekkers, Dr. Hornstra, Dr. Leijten, Prof. Dr. Hoekstra, Dr. Groenman and Ms. Doffer have reported no biomedical financial interests or potential conflicts of interest.

## ETHICAL CONSIDERATIONS

No ethical approval was required for this research review.

## Supporting information

Supporting Information S1

## Data Availability

Data sharing not applicable – no new data generated. Most data is available publicly, as it was extracted from published manuscripts. We received some data (<5%) from the individual authors, therefore this data is not publicly available.

## ENDNOTE

^1^
Note that we used ‘predictor’ instead of ‘moderator’, which is commonly used in meta‐analyses, because we investigated whether these variables were related to within‐ instead of between‐group effect sizes (Kraemer et al., 2002).
