# Supplementary material for: Sustained improvements by behavioural parent training for children with attention‐deficit/hyperactivity disorder: A meta‐analytic review of longer‐term child and parental outcomes
Source: JCPP Adv. 2023 Sep 4;3(3):e12196. doi: 10.1002/jcv2.12196 (PMC10501699; doi:10.1002/jcv2.12196)
Supplement: Supplementary file 1 — Supporting Information S1 [file JCV2-3-e12196-s001.docx]

**Supporting Information**

**Appendix S1 – PRISMA 2009 Checklist**

| **Section/topic** | **#** | **Checklist item** | **Reported on page #** |
| --- | --- | --- | --- |
| **TITLE** | | |  |
| Title | 1 | Identify the report as a systematic review, meta-analysis, or both. | Title page |
| **ABSTRACT** | | |  |
| Structured summary | 2 | Provide a structured summary including, as applicable: background; objectives; data sources; study eligibility criteria, participants, and interventions; study appraisal and synthesis methods; results; limitations; conclusions and implications of key findings; systematic review registration number. | 2-3 |
| **INTRODUCTION** | | |  |
| Rationale | 3 | Describe the rationale for the review in the context of what is already known. | 4-7 |
| Objectives | 4 | Provide an explicit statement of questions being addressed with reference to participants, interventions, comparisons, outcomes, and study design (PICOS). | 7 |
| **METHODS** | | |  |
| Protocol and registration | 5 | Indicate if a review protocol exists, if and where it can be accessed (e.g., Web address), and, if available, provide registration information including registration number. | 8 |
| Eligibility criteria | 6 | Specify study characteristics (e.g., PICOS, length of follow-up) and report characteristics (e.g., years considered, language, publication status) used as criteria for eligibility, giving rationale. | 8-9 |
| Information sources | 7 | Describe all information sources (e.g., databases with dates of coverage, contact with study authors to identify additional studies) in the search and date last searched. | 9  Suppl. 3 |
| Search | 8 | Present full electronic search strategy for at least one database, including any limits used, such that it could be repeated. | Suppl. 3 |
| Study selection | 9 | State the process for selecting studies (i.e., screening, eligibility, included in systematic review, and, if applicable, included in the meta-analysis). | Suppl. 2 |
| Data collection process | 10 | Describe method of data extraction from reports (e.g., piloted forms, independently, in duplicate) and any processes for obtaining and confirming data from investigators. | 9-10 |
| Data items | 11 | List and define all variables for which data were sought (e.g., PICOS, funding sources) and any assumptions and simplifications made. | 9-10 |
| Risk of bias in individual studies | 12 | Describe methods used for assessing risk of bias of individual studies (including specification of whether this was done at the study or outcome level), and how this information is to be used in any data synthesis. | 12 |
| Summary measures | 13 | State the principal summary measures (e.g., risk ratio, difference in means). | 10-11 |
| Synthesis of results | 14 | Describe the methods of handling data and combining results of studies, if done, including measures of consistency (e.g., I^2^) for each meta-analysis. | 12 |

| **Section/topic** | **#** | **Checklist item** | **Reported on page #** |
| --- | --- | --- | --- |
| Risk of bias across studies | 15 | Specify any assessment of risk of bias that may affect the cumulative evidence (e.g., publication bias, selective reporting within studies). | 12 |
| Additional analyses | 16 | Describe methods of additional analyses (e.g., sensitivity or subgroup analyses, meta-regression), if done, indicating which were pre-specified. | 10 |
| **RESULTS** | | |  |
| Study selection | 17 | Give numbers of studies screened, assessed for eligibility, and included in the review, with reasons for exclusions at each stage, ideally with a flow diagram. | 12-13 Suppl. 2 |
| Study characteristics | 18 | For each study, present characteristics for which data were extracted (e.g., study size, PICOS, follow-up period) and provide the citations. | Table 1 |
| Risk of bias within studies | 19 | Present data on risk of bias of each study and, if available, any outcome level assessment (see item 12). | 13  Suppl. 4 |
| Results of individual studies | 20 | For all outcomes considered (benefits or harms), present, for each study: (a) simple summary data for each intervention group (b) effect estimates and confidence intervals, ideally with a forest plot. | Table 1  Table 2 Figure 1  Suppl. 5 |
| Synthesis of results | 21 | Present results of each meta-analysis done, including confidence intervals and measures of consistency. | Table 2 |
| Risk of bias across studies | 22 | Present results of any assessment of risk of bias across studies (see Item 15). | 14  17-18 Suppl. 9-12 |
| Additional analysis | 23 | Give results of additional analyses, if done (e.g., sensitivity or subgroup analyses, meta-regression [see Item 16]). | 18-20  Suppl. 14-15 |
| **DISCUSSION** | | |  |
| Summary of evidence | 24 | Summarize the main findings including the strength of evidence for each main outcome; consider their relevance to key groups (e.g., healthcare providers, users, and policy makers). | 21-26 |
| Limitations | 25 | Discuss limitations at study and outcome level (e.g., risk of bias), and at review-level (e.g., incomplete retrieval of identified research, reporting bias). | 25-26 |
| Conclusions | 26 | Provide a general interpretation of the results in the context of other evidence, and implications for future research. | 21-26 |
| **FUNDING** | | |  |
| Funding | 27 | Describe sources of funding for the systematic review and other support (e.g., supply of data); role of funders for the systematic review. | 38 |

*From:* Moher D, Liberati A, Tetzlaff J, Altman DG, The PRISMA Group (2009). Preferred Reporting Items for Systematic Reviews and Meta-Analyses: The PRISMA Statement. PLoS Med 6(7): e1000097. doi:10.1371/journal.pmed1000097

For more information, visit: **www.prisma-statement.org**.

**Appendix S2 – PRISMA Flowchart**

**Figure S1.1.** *PRISMA flowchart*


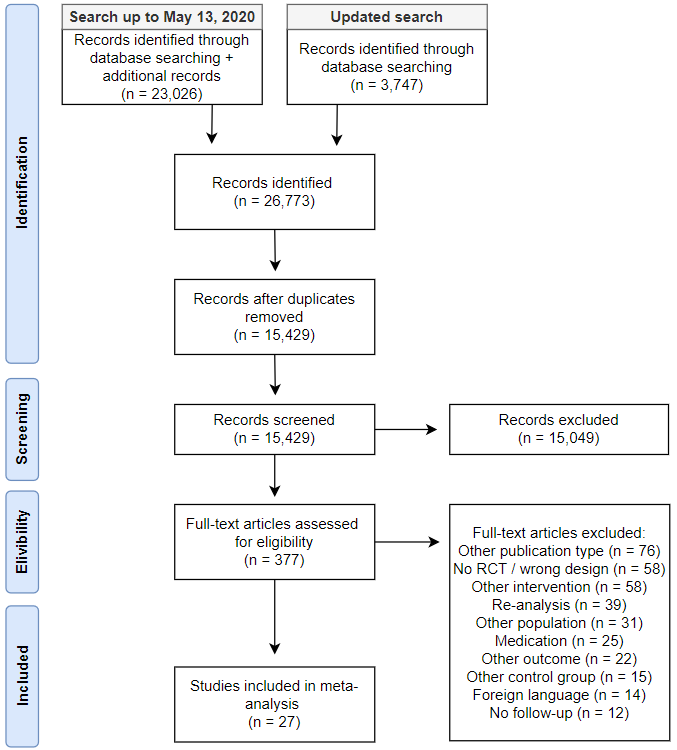


*Note.* RCT = randomized controlled trial.

**Appendix S3 – Search Terms per Database**

**PubMed**

("Attention Deficit Disorder with Hyperactivity"[Mesh] OR ADHD[tiab] OR ADD[tiab] OR attention deficit*[tiab] OR hyperactiv*[tiab] OR hyperkinetic*[tiab] OR minimal brain deficit*[tiab] OR minimal brain dysfunction*[tiab])

**AND**

(("Psychotherapy"[Mesh] OR psychotherap*[tiab] OR psychological therap*[tiab] OR psychological intervent*[tiab] OR psychoeduca*[tiab] OR mentoring*[tiab] OR coaching*[tiab] OR mindful*[tiab] OR relax*[tiab] OR meditat*[tiab])

OR

((parent*[tiab] OR mother[tiab] OR father[tiab] OR teacher*[tiab] OR school*[tiab])

*AND*

(program*[tiab] OR train*[tiab] OR educa*[tiab] OR therapy*[tiab] OR therapies*[tiab] OR therapeu*[tiab]OR intervention*[tiab] OR coaching*[tiab] OR counseling*[tiab]))

OR

((behavio*[tiab] OR cognit*[tiab] OR “acceptance and commitment”[tiab] OR dialectica*[tiab])

*AND*

(program*[tiab] OR therapy*[tiab] OR therapies*[tiab] OR therapeu*[tiab] OR intervention*[tiab] OR treatment*[tiab] OR train*[tiab]))

OR

(psychosocial[tiab]

*AND*

(treatment*[tiab] OR therapy*[tiab] OR therapies*[tiab] OR therapeu*[tiab] OR train*[tiab] OR intervention*[tiab] OR program*[tiab]))

OR

((skill*[tiab] OR organization*[tiab] OR organisation*[tiab] OR planning*[tiab] OR play*[tiab])

*AND*

(train*[tiab] OR intervention*[tiab] OR program*[tiab])))

**AND**

("Adolescent"[Mesh] OR "Child"[Mesh] OR child*[tiab] OR school*[tiab] OR infan*[tiab] OR adolescen*[tiab] OR pediatri*[tiab] OR paediatr*[tiab] OR boy[tiab] OR boys[tiab] OR boyhood[tiab] OR girl[tiab] OR girls[tiab] OR girlhood[tiab] OR youth[tiab] OR youths[tiab] OR teen[tiab] OR teens[tiab] OR teenage*[tiab] OR puberty[tiab] OR preschool*[tiab] OR toddler*[tiab] OR juvenile*[tiab] OR kids[tiab])

**AND**

("Controlled Clinical Trial" [Publication Type] OR "Randomized Controlled Trials as Topic"[Mesh] OR "Follow-Up Studies"[Mesh] OR follow-up[tiab] OR followup[tiab] OR control group*[tiab] OR "Random Allocation"[Mesh] OR random*[tiab] OR trial[ti])

**EMBASE EMBASE.com** (combine separate parts on website)

('attention deficit disorder'/exp OR (ADHD OR ADD OR ‘attention deficit*’

OR hyperactiv* OR hyperkinetic* OR ‘minimal brain deficit*’ OR ‘minimal brain dysfunction*’):ab,ti)

**AND**

('psychotherapy'/exp OR (psychotherap* OR ‘psychological therap*’ OR ‘psychological intervent*’ OR psychoeduca* OR mentoring* OR coaching* OR mindful* OR relax*):ab,ti)

OR

((((parent* OR mother OR father OR teacher* OR school*)

*AND*

(program* OR train* OR educa* OR therapy* OR therapies* OR therapeu*OR intervention* OR coaching* OR counseling*))

OR

((behavio* OR cognit* OR ‘acceptance and commitment’ OR dialectica*)

*AND*

(program* OR therapy* OR therapies* OR therapeu* OR intervention* OR treatment* OR train*))

OR

(psychosocial

*AND*

(treatment* OR therapy* OR therapies* OR therapeu* OR train* OR intervention* OR program*))

OR

((skill* OR organization* OR organisation* OR planning* OR play*)

*AND*

(train* OR intervention* OR program*))):ab,ti)

**AND**

('juvenile'/exp OR (child* OR school* OR infan* OR adolescen* OR pediatri* OR paediatr* OR boy OR boys OR boyhood OR girl OR girls OR girlhood OR youth OR youths OR teen OR teens OR teenage* OR puberty OR preschool* OR toddler* OR juvenile* OR kids):ab,ti)

**AND**

('controlled clinical trial'/exp OR 'randomization'/exp OR (‘control group*’ OR random*):ab,ti OR trial:ti)

**EBSCO PsycINFO** (add separate parts)

(DE "Attention Deficit Disorder" OR DE "Attention Deficit Disorder with Hyperactivity" OR ADHD OR ADD OR “attention deficit*” OR hyperactiv* OR hyperkinetic* OR “minimal brain deficit*” OR “minimal brain dysfunction*”)

**AND**

(DE "Psychotherapy" OR DE "Child Psychotherapy" OR DE "Play Therapy" OR DE "Behavior Therapy" OR DE "Aversion Therapy" OR DE "Conversion Therapy" OR DE "Dialectical Behavior Therapy" OR DE "Exposure Therapy" OR DE "Implosive Therapy" OR DE "Reciprocal Inhibition Therapy" OR DE "Response Cost" OR DE "Systematic Desensitization Therapy" AND DE "Cognitive Behavior Therapy" OR DE "Cognitive Therapy" OR DE "Group Psychotherapy" OR DE "Neurotherapy" OR psychotherap* OR “psychological therap*” OR “psychological intervent*” OR psychoeduca* OR mentoring* OR coaching* OR mindful* OR relax*)

OR

((parent* OR mother OR father OR teacher* OR school*)

*AND*

(program* OR train* OR educa* OR therapy* OR therapies* OR therapeu*OR intervention* OR coaching* OR counseling*))

OR

((behavio* OR cognit* OR ‘acceptance and commitment’ OR dialectica*)

*AND*

(program* OR therapy* OR therapies* OR therapeu* OR intervention* OR treatment* OR train*))

OR

(psychosocial

*AND*

(treatment* OR therapy* OR therapies* OR therapeu* OR train* OR intervention* OR program*))

OR

((skill* OR organization* OR organisation* OR planning* OR play*)

*AND*

(train* OR intervention* OR program*))

**AND**

(AG (childhood OR adolescence) OR (child* OR school* OR infan* OR adolescen* OR pediatri* OR paediatr* OR boy OR boys OR boyhood OR girl OR girls OR girlhood OR youth OR youths OR teen OR teens OR teenage* OR puberty OR preschool* OR toddler* OR juvenile* OR kids))

**AND**

(“control group*” OR random* OR "controlled trial" OR "controlled study" OR "experimental study" OR "experimental design" OR TI trial)

**EBSCO CINAHL** (separate parts)

(MH "Attention Deficit Hyperactivity Disorder" OR "Attention Deficit Disorder with Hyperactivity" OR ADHD OR ADD OR “attention deficit*” OR hyperactiv* OR hyperkinetic* OR “minimal brain deficit*” OR “minimal brain dysfunction*”)

**AND**

(MH "Psychotherapy+" OR psychotherap* OR “psychological therap*” OR “psychological intervent*” OR psychoeduca* OR mentoring* OR coaching* OR mindful* OR relax*)

OR

((parent* OR mother OR father OR teacher* OR school*)

*AND*

(program* OR train* OR educa* OR therapy* OR therapies* OR therapeu*OR intervention* OR coaching* OR counseling*))

OR

((behavio* OR cognit* OR ‘acceptance and commitment’ OR dialectica*)

*AND*

(program* OR therapy* OR therapies* OR therapeu* OR intervention* OR treatment* OR train*))

OR

(psychosocial

*AND*

(treatment* OR therapy* OR therapies* OR therapeu* OR train* OR intervention* OR program*))

OR

((skill* OR organization* OR organisation* OR planning* OR play*)

*AND*

(train* OR intervention* OR program*))

**AND**

(AG child OR (child* OR school* OR infan* OR adolescen* OR pediatri* OR paediatr* OR boy OR boys OR boyhood OR girl OR girls OR girlhood OR youth OR youths OR teen OR teens OR teenage* OR puberty OR preschool* OR toddler* OR juvenile* OR kids))

**AND**

(MH "Clinical Trials+" OR “control group*” OR random* OR "controlled trial" OR "controlled study" OR "experimental study" OR "experimental design" OR TI trial)

**EBSCO ERIC** (separate parts)

(DE "Attention Deficit Disorder" OR DE "Attention Deficit Hyperactivity Disorder" OR ADHD OR ADD OR “attention deficit*” OR hyperactiv* OR hyperkinetic* OR “minimal brain deficit*” OR “minimal brain dysfunction*”)

**AND**

(DE "Psychotherapy" OR psychotherap* OR “psychological therap*” OR “psychological intervent*” OR psychoeduca* OR mentoring* OR coaching* OR mindful* OR relax*)

OR

((parent* OR mother OR father OR teacher* OR school*)

*AND*

(program* OR train* OR educa* OR therapy* OR therapies* OR therapeu*OR intervention* OR coaching* OR counseling*))

OR

((behavio* OR cognit* OR ‘acceptance and commitment’ OR dialectica*)

*AND*

(program* OR therapy* OR therapies* OR therapeu* OR intervention* OR treatment* OR train*))

OR

(psychosocial

*AND*

(treatment* OR therapy* OR therapies* OR therapeu* OR train* OR intervention* OR program*))

OR

((skill* OR organization* OR organisation* OR planning* OR play*)

*AND*

(train* OR intervention* OR program*))

**AND**

(child* OR school* OR infan* OR adolescen* OR pediatri* OR paediatr* OR boy OR boys OR boyhood OR girl OR girls OR girlhood OR youth OR youths OR teen OR teens OR teenager* OR puberty OR preschool* OR toddler* OR juvenile* OR kids))

**AND**

(“control group*” OR random* OR "controlled trial" OR "controlled study" OR "experimental study" OR "experimental design" OR TI trial)

**Web of Science** (via advanced search**)**

(TS=("Attention Deficit Disorder with Hyperactivity" OR ADHD OR ADD OR “attention deficit*” OR hyperactiv* OR hyperkinetic* OR “minimal brain deficit*” OR “minimal brain dysfunction*”))

**AND**

(TS=(psychotherap* OR psychotherap* OR “psychological therap*” OR “psychological intervent*” OR psychoeduca* OR mentoring* OR coaching* OR mindful* OR relax*

OR

((parent* OR mother OR father OR teacher* OR school*)

*AND*

(program* OR train* OR educa* OR therapy* OR therapies* OR therapeu*OR intervention* OR coaching* OR counseling*))

OR

((behavio* OR cognit* OR ‘acceptance and commitment’ OR dialectica*)

*AND*

(program* OR therapy* OR therapies* OR therapeu* OR intervention* OR treatment* OR train*))

OR

(psychosocial

*AND*

(treatment* OR therapy* OR therapies* OR therapeu* OR train* OR intervention* OR program*))

OR

((skill* OR organization* OR organisation* OR planning* OR play*)

*AND*

(train* OR intervention* OR program*))))

**AND**

(TS=(child* OR school* OR infan* OR adolescen* OR pediatri* OR paediatr* OR boy OR boys OR boyhood OR girl OR girls OR girlhood OR youth OR youths OR teen OR teens OR teenager* OR puberty OR preschool* OR toddler* OR juvenile* OR kids))

**AND**

(TS=(“clinical trial*” OR “control group*” OR random* OR "controlled trial" OR "controlled study" OR "experimental study" OR "experimental design"))

**Appendix S4 – Risk of Bias Assessment**

**Figure S2.1.** *Risk of bias graph*


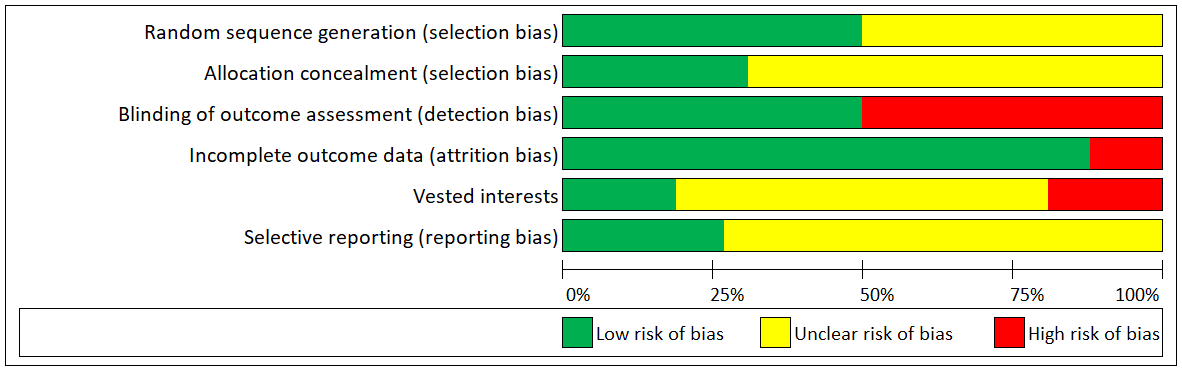


**Figure S2.2.** *Risk of bias summary*

|  |  | Random sequence generation | Allocation concealment | Blinding of outcome assessment | Incomplete outcome data | Vested interests | Selective outcome reporting |
| --- | --- | --- | --- | --- | --- | --- | --- |
| Abikoff | 2015 | **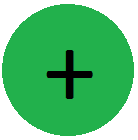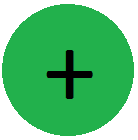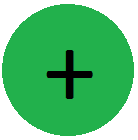** |  | **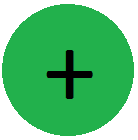** |  | **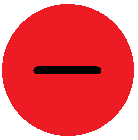** | **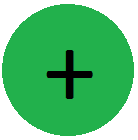** |
| Au | 2014 | **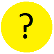** | **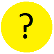** | **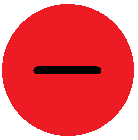** | **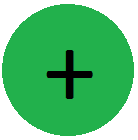** | **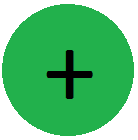** | **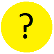** |
| Chacko | 2009 | **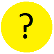** | **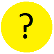** | **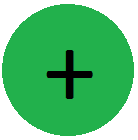** | **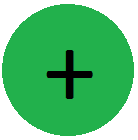** | **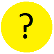** | **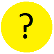** |
| Chesterfield | 2021 | **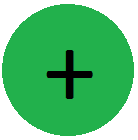** | **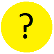** | **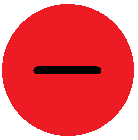** | **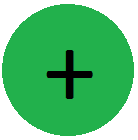** | **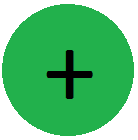** | **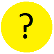** |
| Ferrin | 2014 | **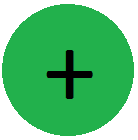** | **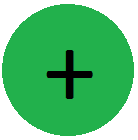** | **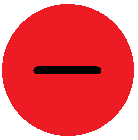** | **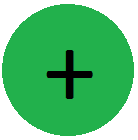** | **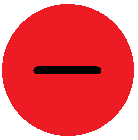** | **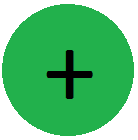** |
| Ferrin | 2016 | **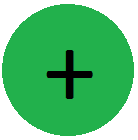** | **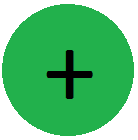** | **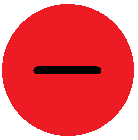** | **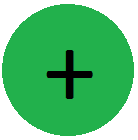** | **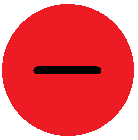** | **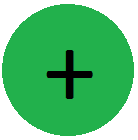** |
| Franke | 2016 | **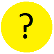** | **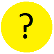** | **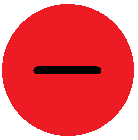** | **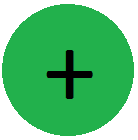** | **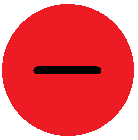** | **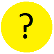** |
| Hoath | 2002 | **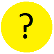** | **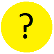** | **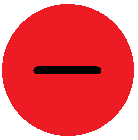** | **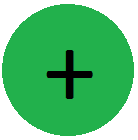** | **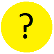** | **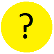** |
| Jiang | 2018 | **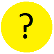** | **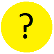** | **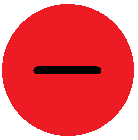** | **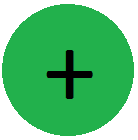** | **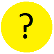** | **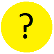** |
| Lange | 2018 | **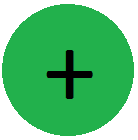** | **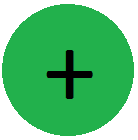** | **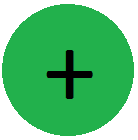** | **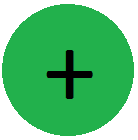** | **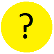** | **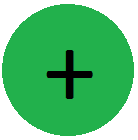** |
| Larsen | 2021 | **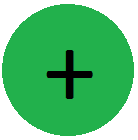** | **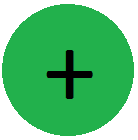** | **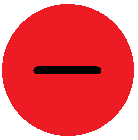** | **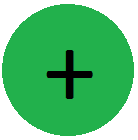** | **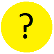** | **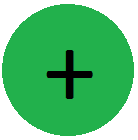** |
| Matos | 2009 | **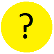** | **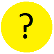** | **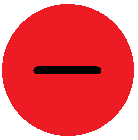** | **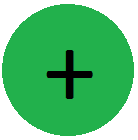** | **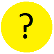** | **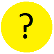** |
| Mautone | 2012 | **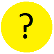** | **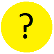** | **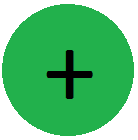** | **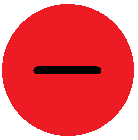** | **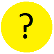** | **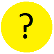** |
| Mikami | 2020 | **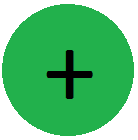** | **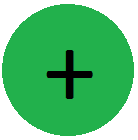** | **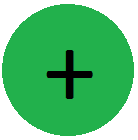** | **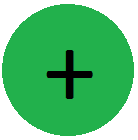** | **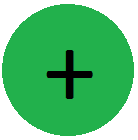** | **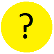** |
| Pfiffner | 2014 | **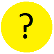** | **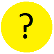** | **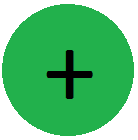** | **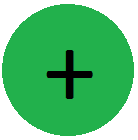** | **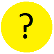** | **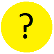** |
| Pisterman | 1989 | **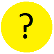** | **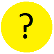** | **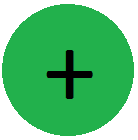** | **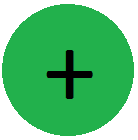** | **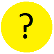** | **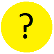** |
| Pisterman | 1992a | **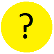** | **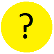** |  |  |  |  |
| Pisterman | 1992b | ***** |  |  |  |  |  |
| Power | 2012 |  |  |  |  |  |  |
| Sibley | 2016 |  |  |  |  |  |  |
| Sibley | 2021 |  |  |  |  |  |  |
| Smit | 2022 |  |  |  |  |  |  |
| Sonuga-Barke | 2001 |  |  |  |  |  |  |
| Sonuga-Barke | 2004 |  |  |  |  |  |  |
| Sonuga-Barke | 2018 |  |  |  |  |  |  |
| Thompson | 2009 |  |  |  |  |  |  |
| Van den Hoofdakker | 2007 |  |  |  |  |  |  |

*Note.* *Pisterman et al. (1992b) was not scored separately, as it is a combined dataset of

Pisterman et al. (1989) and Pisterman et al. (1992a).

**Appendix S5 – Forest Plots**

**Figure S3.1.** *Forest plot of within-group effect sizes from pre-intervention to follow-up measurement on all outcome measures for both primary outcomes*

(a) ADHD symptoms

*Note.* BASC-PRS = Behavioural Assessment System for Children – Parent Rating Scale; BPT = Behavioural Parent Training; C3RS = Conners-3 Parent Short Form Rating Scale; CLAS = Child Life and Attention Skills; CPRS = Conners Parent Rating Scale; CSI = Child Symptom Inventory; CTRS = Conners Teacher Rating Scale; DBD = Disruptive Behaviours Disorders rating scale; DBRS = Disruptive Behaviour Rating Scale; DSM = Diagnostic and Statistical Manual of Mental Disorders; H = hyperactivity; HNC = Helping the Noncompliant Child; I = impulsivity; IN = inattention; IV = 4^th^ edition; IY = Incredible Years; NFPP = New Forest Parenting Programme; P = parent; PACS = Parental Account of Childhood Symptoms; PFT = Parent-Focused Treatment; R = restlessness; RS = rating scale; SNAP = Swanson, Nolan, and Pelham Questionnaire; STEPP = Strategies to Enhance Positive Parenting; T = teacher; WWP = Werry-Weiss-Peters-Hyperactivity scale.

(b) Behavioural problems

*Note.* AAPC = Adolescent Academic Problems Checklist; A = aggression; BASC-PRS = Behavioural Assessment System for Children – Parent Rating Scale; BCL = Behaviour Check List; BPT = Behavioural Parent Training; CBCL = Child Behaviour Checklist; CHQ-PF28-BE = Child Health Questionnaire Parent Form 28 – Behaviour subscale; DBD = Disruptive Behaviours Disorders rating scale; DBRS = Disruptive Behaviour Rating Scale; D = defiance; ECBI = Eyberg Child Behaviour Inventory; IY = Incredible Years; NFPP = New Forest Parenting Programme; HNC = Helping the Noncompliant Child; NYPRS = New York Parent Rating Scale; NYTRS = New York Teacher Rating Scale; ODD = oppositional defiant disorder; P = parent; PACS = Parental Account of Childhood Symptoms; SDQ = Strengths and Difficulties Questionnaire; SNAP = Swanson, Nolan, and Pelham Questionnaire; STEPP = Strategies to Enhance Positive Parenting; T = teacher.

**Figure S3.2.** *Forest plot of within-group effect sizes from post-intervention to follow-up measurement on all outcome measures for both primary outcomes*

(a) ADHD symptoms

*Note.* BASC-PRS = Behavioural Assessment System for Children – Parent Rating Scale; BPT = Behavioural Parent Training; C3RS = Conners-3 Parent Short Form Rating Scale; CLAS = Child Life and Attention Skills; CPRS = Conners Parent Rating Scale; CSI = Child Symptom Inventory; CTRS = Conners Teacher Rating Scale; DBD = Disruptive Behaviours Disorders rating scale; DBRS = Disruptive Behaviour Rating Scale; DSM = Diagnostic and Statistical Manual of Mental Disorders; H = hyperactivity; HNC = Helping the Noncompliant Child; I = impulsivity; IN = inattention; IV = 4^th^ edition; IY = Incredible Years; NFPP = New Forest Parenting Programme; P = parent; PACS = Parental Account of Childhood Symptoms; PFT = Parent-Focused Treatment; R = restlessness; RS = rating scale; SNAP = Swanson, Nolan, and Pelham Questionnaire; STEPP = Strategies to Enhance Positive Parenting; T = teacher; WWP = Werry-Weiss-Peters-Hyperactivity scale.

(b) Behavioural problems

*Note.* AAPC = Adolescent Academic Problems Checklist; A = aggression; BASC-PRS = Behavioural Assessment System for Children – Parent Rating Scale; BCL = Behaviour Check List; BPT = Behavioural Parent Training; CBCL = Child Behaviour Checklist; CHQ-PF28-BE = Child Health Questionnaire Parent Form 28 – Behaviour subscale; DBD = Disruptive Behaviours Disorders rating scale; DBRS = Disruptive Behaviour Rating Scale; D = defiance; ECBI = Eyberg Child Behaviour Inventory; IY = Incredible Years; NFPP = New Forest Parenting Programme; HNC = Helping the Noncompliant Child; NYPRS = New York Parent Rating Scale; NYTRS = New York Teacher Rating Scale; ODD = oppositional defiant disorder; P = parent; PACS = Parental Account of Childhood Symptoms; SDQ = Strengths and Difficulties Questionnaire; SNAP = Swanson, Nolan, and Pelham Questionnaire; STEPP = Strategies to Enhance Positive Parenting; T = teacher.

**Figure S3.3.** *Forest plot of between-group effect sizes from post-intervention to follow-up measurement on all outcome measures for both primary outcomes*

(a) ADHD symptoms

*Note.* BCL = Behaviour Check List; BPT = Behavioural Parent Training; C3RS = Conners-3 Parent Short Form Rating Scale; CLAS = Child Life and Attention Skills; CPRS = Conners Parent Rating Scale; CSI = Child Symptom Inventory; CTRS = Conners Teacher Rating Scale; DBD = Disruptive Behaviours Disorders rating scale; DSM = Diagnostic and Statistical Manual of Mental Disorders; H = hyperactivity; I = impulsivity; IN = inattention; IV = 4^th^ edition; P = parent; PACS = Parental Account of Childhood Symptoms; PFT = Parent-Focused Treatment; R = restlessness; RS = rating scale; SNAP = Swanson, Nolan, and Pelham Questionnaire; STEPP = Strategies to Enhance Positive Parenting; T = teacher; WWP = Werry-Weiss-Peters-Hyperactivity scale.

(b) Behavioural problems

*Note.* AAPC = Adolescent Academic Problems Checklist; A = aggression; BCL = Behaviour Check List; BPT = Behavioural Parent Training; CBCL = Child Behaviour Checklist; CHQ-PF28-BE = Child Health Questionnaire Parent Form 28 – Behaviour subscale; CPRS = Conners Parent Rating Scale; DBD = Disruptive Behaviours Disorders rating scale; D = defiance; ODD = oppositional defiant disorder; P = parent; PACS = Parental Account of Childhood Symptoms; SDQ = Strengths and Difficulties Questionnaire; SNAP = Swanson, Nolan, and Pelham Questionnaire; STEPP = Strategies to Enhance Positive Parenting; T = teacher.

**Appendix S6 – Sensitivity Analyses: Within-Group**

**Table S1.1.** *Within-group effect sizes per outcome domain using the SMCC with correlation values of r = .30, r = .50 and r = .70*

|  |  | **Pre – Follow-up** | | **Post – Follow-up** | | **Pre – Post** | |
| --- | --- | --- | --- | --- | --- | --- | --- |
|  | Correlation | ES | *p* | ES | *p* | ES | *p* |
| **ADHD Symptoms** | *r* = .30 | .453 | .000 | .056 | .038 | .451 | .000 |
|  | *r* = .50 | .530 | .000 | .062 | .050 | .530 | .000 |
|  | *r* = .70 | .670 | .000 | .073 | .066 | .673 | .000 |
| **Behavioural Problems** | *r* = .30 | .403 | .000 | .017 | .541 | .403 | .000 |
|  | *r* = .50 | .472 | .000 | .015 | .681 | .475 | .000 |
|  | *r* = .70 | .596 | .000 | .013 | .802 | .603 | .000 |
| **Positive Parenting** | *r* = .30 | .559 | .000 | -.058 | .123 | .596 | .000 |
|  | *r* = .50 | .663 | .000 | -.062 | .152 | .704 | .000 |
|  | *r* = .70 | .852 | .000 | -.069 | .213 | .899 | .000 |
| **Negative Parenting** | *r* = .30 | .673 | .000 | .008 | .838 | .674 | .000 |
|  | *r* = .50 | .793 | .000 | .010 | .804 | .787 | .000 |
|  | *r* = .70 | 1.005 | .000 | .001 | .992 | .985 | .000 |
| **Parenting Sense of**  **Competence** | *r* = .30 | .463 | .001 | -.026 | .440 | .579 | .000 |
|  | *r* = .50 | .544 | .001 | -.030 | .379 | .678 | .000 |
|  | *r* = .70 | .693 | .000 | -.043 | .375 | .860 | .000 |
| **Parent-Child Relationship**  **Quality** | *r* = .30 | .289 | .048 | -.002 | .988 | .314 | .005 |
|  | *r* = .50 | .343 | .049 | -.022 | .885 | .377 | .006 |
|  | *r* = .70 | .405 | .039 | -.024 | .900 | .487 | .006 |
| **Parental Mental Health** | *r* = .30 | .338 | .002 | -.008 | .847 | .386 | .000 |
|  | *r* = .50 | .394 | .002 | -.010 | .843 | .447 | .000 |
|  | *r* = .70 | .496 | .002 | -.012 | .854 | .559 | .000 |

*Note.* Effect sizes (ES) represent standardized mean change scores using change score standardization with a correlation of *r* = .50.

**Appendix S7 – Within-Group Effects Control Condition**

**Table S2.1.** *Within-group effects of the control condition per outcome domain*

|  |  | ***k*** | ***n-es*** | **ES** | **95% CI** | ***I^2^* (level 2, %)** | ***I^2^* (level 3, %)** |
| --- | --- | --- | --- | --- | --- | --- | --- |
| **ADHD symptoms** | Within pre – follow-up | 16^+^ | 46^+^ | .23^**^ | .07 – .39 | 31.7 | 52.7 |
|  | Within post – follow-up | 15 | 43 | .09^*^ | .01 – .17 | 0.0 | 43.6 |
|  | Within pre – post ^1^ | 15 | 43 | .21^***^ | .10 – .31 | 26.1 | 41.5 |
|  | Within pre – post ^2^ | 22 | 69 | .18^***^ | .10 – .25 | 29.4 | 29.1 |
| **Behavioural problems** | Within pre – follow-up | 17 | 24 | .18^**^ | .08 – .28 | 61.0 | 0.0 |
|  | Within post – follow-up | 17 | 24 | .07^*^ | .00 – .13 | 0.0 | 6.5 |
|  | Within pre – post ^1^ | 17 | 24 | .10^**^ | .03 – .16 | 4.6 | 0.0 |
|  | Within pre – post ^2^ | 26 | 52 | .12^***^ | .07 – .17 | 0.0 | 5.3 |
| **Positive parenting** | Within pre – follow-up | 9 | 16 | .08 | -.04 – .19 | 38.2 | 0.0 |
|  | Within post – follow-up | 9 | 16 | .06 | -.05 – .16 | 0.0 | 21.8 |
|  | Within pre – post ^1^ | 9 | 16 | .01 | -.13 – .14 | 54.3 | 0.0 |
|  | Within pre – post ^2^ | 14 | 21 | -.04 | -.17 – .08 | 58.0 | 2.4 |
| **Negative parenting** | Within pre – follow-up | 8 | 17 | .43^***^ | .28 – .59 | 63.1 | 0.0 |
|  | Within post – follow-up | 8 | 17 | .15^**^ | .05 – .25 | 0.0 | 12.8 |
|  | Within pre – post ^1^ | 8 | 17 | .29^***^ | .17 – .40 | 37.0 | 0.0 |
|  | Within pre – post ^2^ | 9 | 20 | .27^***^ | .16 – .38 | 32.9 | 0.0 |
| **Parenting sense of competence** | Within pre – follow-up | 9^+^ | 16^+^ | .25 | -.09 – .59 | 0.0 | 89.6 |
|  | Within post – follow-up | 8 | 14 | .10^*^ | .02 – .18 | 0.0 | 0.0 |
|  | Within pre – post ^1^ | 8 | 14 | .24^*^ | .01 – .47 | 0.0 | 77.1 |
|  | Within pre – post ^2^ | 12 | 19 | .19^*^ | .02 – .36 | 0.0 | 67.9 |
| **Parent-child relationship quality** | Within pre – follow-up | 8 | 11 | .22 | -.38 – .81 | 0.0 | 96.7 |
|  | Within post – follow-up | 8 | 11 | .24 | -.47 – .94 | 0.0 | 97.7 |
|  | Within pre – post ^1^ | 8 | 11 | .03 | -.05 – .12 | 0.0 | 0.0 |
|  | Within pre – post ^2^ | 10 | 13 | .02 | -.06 – .10 | 0.0 | 0.0 |
| **Parental mental health** | Within pre – follow-up | 9^+^ | 13^+^ | .05 | -.09 – .20 | 50.0 | 0.0 |
|  | Within post – follow-up | 8 | 12 | -.02 | -.15 – .12 | 19.1 | 14.6 |
|  | Within pre – post ^1^ | 8 | 12 | .11 | -.03 – .25 | 39.1 | 0.0 |
|  | Within pre – post ^2^ | 13 | 17 | .11^*^ | .01 – .20 | 20.0 | 0.0 |

*Note*: *k* = number of studies, *n-es* = number of effect sizes; CI = Confidence Interval. Effect sizes (ES) represent standardized mean change scores using change score standardization with a correlation of *r* = .50, with 95% confidence intervals. Positive effect sizes indicate beneficial effects of the intervention for all outcomes. *I^2^* represents between-variables and between-studies heterogeneity (level 2 and 3 respectively).

****p* < .001, ***p* < .01, **p* < .05.

^1^: Data of all studies that conducted a follow-up measurement for the control group, to compare pre – post with pre – follow-up and post – follow-up for the control group.

^2^: Data of all studies that measured pre – post for the control group, to compare the pre – post data for the intervention and the control group.

^+^: Sonuga-Barke et al. (2004) only reported pre-measurement and later follow-up, and therefore the number of studies and effect sizes differs from the other comparisons.

**Appendix S8 – Patterns of Change**

**Table S3.1.** *Meta-regression analyses on the difference between pre-post and pre-fu between-group effect sizes*

|  |  | ***k*(*n-es*)** | $\hat{\boldsymbol{\beta}}$***_0_*** | $\hat{\boldsymbol{\beta}}$***_1_*** | ***I^2^* (level 2, 3)** |
| --- | --- | --- | --- | --- | --- |
| **ADHD Symptoms** | Pre – Post | 15 (43) | .28^***^ |  | 2.4, 64.5 |
|  | Pre – Follow-up | 16 (46) | .24^**^ |  |  |
|  | Comparison |  |  | -.04 |  |
| **Behavioural Problems** | Pre – Post | 17 (24) | .20^***^ |  | 27.8, 1.6 |
|  | Pre – Follow-up | 17 (24) | .15^**^ |  |  |
|  | Comparison |  |  | -.05 |  |
| **Positive Parenting** | Pre – Post | 9 (16) | .71^***^ |  | 49.1, 36.5 |
|  | Pre – Follow-up | 9 (16) | .57^**^ |  |  |
|  | Comparison |  |  | -.14 |  |
| **Negative Parenting** | Pre – Post | 8 (17) | .43^*^ |  | 34.1, 51.6 |
|  | Pre – Follow-up | 8 (17) | .25 |  |  |
|  | Comparison |  |  | -.18 |  |
| **Parenting Sense of Competence** | Pre – Post | 8 (14) | .50^***^ |  | 0.0, 71.5 |
|  | Pre – Follow-up | 9 (16) | .38^**^ |  |  |
|  | Comparison |  |  | -.12 |  |
| **Parent-Child Relationship Quality** | Pre – Post | 8 (11) | .27^*^ |  | 8.9, 47.5 |
|  | Pre – Follow-up | 8 (11) | .25^*^ |  |  |
|  | Comparison |  |  | -.02 |  |
| **Parental Mental Health** | Pre – Post | 8 (12) | .23 |  | 14.8, 48.4 |
|  | Pre – Follow-up | 9 (13) | .23 |  |  |
|  | Comparison |  |  | .00 |  |

*Note. k* = number of studies; *n-es* = number of effect sizes; $\hat{\beta}$*_0_* denotes the SMD (in terms of Hedges’ g) for the category referred to in the first column; $\hat{\beta}$*_1_* denotes the change in SMD when the moderator increases with one; I^2^ represents between-variables and between-studies heterogeneity (level 2 and 3 respectively).
Pre – Follow-up effect sizes are coded as 0 and Pre – Post effect sizes as 1, therefore a significant positive $\hat{\beta}$*_1_* would represent a sleeper effect and a significant negative $\hat{\beta}$*_1_* a fade-out effect.

*** *p* < .001, ** *p* < .01, * *p* < .05.

**Table S3.2.** *Meta-regression analysis on the difference between pre-post and pre-fu within-group effect sizes*

|  |  | ***k*(*n*-es)** | $\hat{\boldsymbol{\beta}}$***_0_*** | $\hat{\boldsymbol{\beta}}$***_1_*** | ***I^2^* (level 2, 3)** |
| --- | --- | --- | --- | --- | --- |
| **ADHD Symptoms** | Pre – Post | 22 (69) | .50^***^ |  | 43.2, 46.4 |
|  | Pre – Follow-up | 23 (72) | .55^***^ |  |  |
|  | Comparison |  |  | .05 |  |
| **Behavioural Problems** | Pre – Post | 26 (52) | .47^***^ |  | 17.7, 68.3 |
|  | Pre – Follow-up | 26 (52) | .49^***^ |  |  |
|  | Comparison |  |  | .02 |  |
| **Positive Parenting** | Pre – Post | 14 (21) | .67^***^ |  | 70.7, 22.7 |
|  | Pre – Follow-up | 14 (21) | .63^***^ |  |  |
|  | Comparison |  |  | -.04 |  |
| **Negative Parenting** | Pre – Post | 9 (20) | .76^***^ |  | 45.6, 35.3 |
|  | Pre – Follow-up | 9 (20) | .73^***^ |  |  |
|  | Comparison |  |  | -.03 |  |
| **Parenting Sense of Competence** | Pre – Post | 12 (19) | .60^***^ |  | 0.0, 88.7 |
|  | Pre – Follow-up | 13 (21) | .55^***^ |  |  |
|  | Comparison |  |  | -.05 |  |
| **Parent-Child Relationship Quality** | Pre – Post | 10 (13) | .39^**^ |  | 71.9, 18.0 |
|  | Pre – Follow-up | 10 (13) | .31^*^ |  |  |
|  | Comparison |  |  | -.08 |  |
| **Parental Mental Health** | Pre – Post | 13 (17) | .40^***^ |  | 0.0, 83.8 |
|  | Pre – Follow-up | 14 (18) | .40^***^ |  |  |
|  | Comparison |  |  | .00 |  |

*Note. k* = number of studies; *n-es* = number of effect sizes; $\hat{\beta}$*_0_* denotes the SMCC (in terms of Hedges’ g) for the category referred to in the first column; $\hat{\beta}$*_1_* denotes the change in SMCC when the moderator increases with one; I^2^ represents between-variables and between-studies heterogeneity (level 2 and 3 respectively).
Pre – Follow-up effect sizes are coded as 0 and Pre – Post effect sizes as 1, therefore a significant positive $\hat{\beta}$*_1_* would represent a sleeper effect and a significant negative $\hat{\beta}$*_1_* a fade-out effect.

*** *p* < .001, ** *p* < .01, * *p* < .05.

**Appendix S9 – Robustness Analyses: Funnel Plots**

**Figure S4.1.** *Funnel plot for ADHD symptoms*

*Note*. Grey dots indicate effect sizes of studies included in the meta-analyses, white dots

represent hypothetical studies to make the funnel symmetrical.

**Figure S4.2.** *Funnel plot for behavioural problems*

*Note*. Grey dots indicate effect sizes of studies included in the meta-analyses, white dots

represent hypothetical studies to make the funnel symmetrical.

**Figure S4.3.** *Funnel plot for positive parenting*

*Note*. Grey dots indicate effect sizes of studies included in the meta-analyses, white dots

represent hypothetical studies to make the funnel symmetrical.

**Figure S4.4.** *Funnel plot for negative parenting*

*Note*. Grey dots indicate effect sizes of studies included in the meta-analyses, white dots

represent hypothetical studies to make the funnel symmetrical.

**Figure S4.5.** *Funnel plot for parenting sense of competence*

*Note*. Grey dots indicate effect sizes of studies included in the meta-analyses, white dots

represent hypothetical studies to make the funnel symmetrical.

**Figure S4.6.** *Funnel plot for parent-child relationship quality*

*Note*. Grey dots indicate effect sizes of studies included in the meta-analyses, white dots

represent hypothetical studies to make the funnel symmetrical.

**Figure S4.7.** *Funnel plot for parental mental health*

*Note*. Grey dots indicate effect sizes of studies included in the meta-analyses, white dots

represent hypothetical studies to make the funnel symmetrical.

**Appendix S10 – Robustness Analyses: Trim-and-Fill**

**Table S4.1.** *Within-group effect sizes from pre-intervention to follow-up after
publication bias correction.*

|  |  | ***n-es*** | **ES** | **95% CI** |
| --- | --- | --- | --- | --- |
| **ADHD Symptoms** | Reported effect | 72 | .53^***^ | .40 – .66 |
|  | After trim-fill correction | 77 (+5) | .51^***^ | .41 – .62 |
| **Behavioural Problems** | Reported effect | 52 | .47^***^ | .33 – .61 |
|  | After trim-fill correction | 58 (+6) | .43^***^ | .28 – .58 |
| **Positive Parenting** | Reported effect | 21 | .66^**^ | .38 – .95 |
|  | After trim-fill correction | 26 (+5) | .39^*^ | .01 – .76 |
| **Negative Parenting** | Reported effect | 20 | .79^***^ | .57 – 1.02 |
|  | After trim-fill correction | 26 (+6) | .58^***^ | .28 – .89 |
| **Parenting Sense of Competence** | Reported effect | 21 | .54^***^ | .27 – .82 |
|  | After trim-fill correction | 22 (+1) | .48^***^ | .25 – .72 |
| **Parental Mental Health** | Reported effect | 18 | .39^**^ | .16 – .63 |
|  | After trim-fill correction | 21 (+3) | .34^**^ | .14 – .54 |

*Note. n-es* = number of effect sizes (with added effect sizes to correct for publication bias in parentheses); CI = Confidence Interval. Effect sizes (ES) represent standardized mean change scores using change score standardization with a correlation of *r* = .50. Reported effects represent effects from main manuscript, corrected effects represent effects corrected for publication bias using the trim-and-fill method. Outcome domain parent-child relationship quality is not displayed as there was no publication bias in this domain.

****p* < .001, ***p* < .01, **p* < .05.

**Appendix S11 – Robustness Analyses: P-Curves**

**Figure S5.1.** *P-Curve for ADHD symptoms*

**Figure S5.2.** *P-Curve for behavioural problems*

**Figure S5.3.** *P-Curve for positive parenting*

**Figure S5.4.** *P-Curve for negative parenting*

**Figure S5.5.** *P-Curve for parenting sense of competence*

**Figure S5.6.** *P-Curve for parent-child relationship quality*

**Figure S5.7.** *P-Curve for parenting mental health*

**Appendix S12 – Robustness Analyses: Leave-one-out**

**Table S5.1.** *Leave-one-out analyses for all outcome measures on within-group effect sizes from pre-intervention to follow-up*

|  | **ADHD** | **Behavioural problems** | **Positive parenting** | **Negative parenting** | **Parenting competence** | **Parent-child relationship** | **Parent mental health** |
| --- | --- | --- | --- | --- | --- | --- | --- |
| **ES without leave-one-out** | **.53^***^** | **.47^***^** | **.66^***^** | **.79^***^** | **.54^***^** | **.34^*^** | **.39^**^** |
| Abikoff et al. 2015: HNC | .50^***^ | .47^***^ | .65^***^ | - | .53^**^ | .37 | - |
| Abikoff et al. 2015: NFPP | .52^***^ | .47^***^ | .66^***^ | - | .56^***^ | .38 | - |
| Au et al. 2014 | - | .48^***^ | - | - | .58^***^ | - | - |
| Chacko et al. 2009: BPT | .54^***^ | .49^***^ | - | - | - | .40^*^ | - |
| Chacko et al. 2009: STEPP | .54^***^ | .48^***^ | - | - | - | .35 | - |
| Chesterfield et al. 2021 | .52^***^ | .46^***^ | - | - | - | - | .37^**^ |
| Ferrin et al. 2014 | .55^***^ | .49^***^ | - | - | - | - | .41^**^ |
| Ferrin et al. 2016 | .55^***^ | .49^***^ | - | - | - | - | .46^***^ |
| Franke et al. 2016 | .52^***^ | .47^***^ | - | .82^***^ | .48^**^ | .18^**^ | .36^**^ |
| Hoath et al. 2002 | - | .45^***^ | - | .80^***^ | .53^**^ | - | - |
| Jiang et al. 2018: CLAS | - | - | .69^***^ | .82^***^ | .55^**^ | - | - |
| Jiang et al. 2018: PFT | - | - | .69^***^ | .82^***^ | .56^**^ | - | - |
| Lange et al. 2018 | .54^***^ | .48^***^ | .69^***^ | - | .59^***^ | - | - |
| Larsen et al. 2021 | - | .48^***^ | - | - | - | - | .41^**^ |
| Matos et al. 2009 | .50^***^ | .42^***^ | .64^***^ | - | - | - | .36^**^ |
| Mautone et al. 2012 | .53^***^ | .48^***^ | .72^***^ | .85^***^ | .55^**^ | - | - |
| Mikami et al. 2020 | - | .46^***^ | - | - | - | - | - |
| Pfiffner et al. 2014: CLAS | .51^***^ | - | - | - | - | - | - |
| Pfiffner et al. 2014: PFT | .52^***^ | - | - | - | - | - | - |
| Pisterman et al. 1989 | - | .46^***^ | .54^***^ | .65^***^ | - | - | - |
| Pisterman et al. 1992a | .55^***^ | .47^***^ | .54^***^ | .72^***^ | - | - | - |
| Pisterman et al. 1992b | - | - | - | - | .49^**^ | - | .38^**^ |
| Power et al. 2012 | .53^***^ | .48^***^ | - | .79^***^ | .50^**^ | .38 | - |
| Sibley et al. 2016 | .53^***^ | .49^***^ | .71^***^ | - | - | .39 | .37^**^ |
| Sibley et al. 2021 | .53^***^ | .49^***^ | - | - | - | .38^*^ | - |
| Smit et al. 2022 | - | .47^***^ | .68^***^ | .83^***^ | - | .36 | - |
| Sonuga-Barke et al. 2001 | .54^***^ | .47^***^ | - | - | .56^**^ | - | .40^**^ |
| Sonuga-Barke et al. 2004 | .56^***^ | - | - | - | .63^***^ | - | .46^***^ |
| Sonuga-Barke et al. 2018: NFPP | .54^***^ | .48^***^ | .70^***^ | - | - | - | .38^**^ |
| Sonuga-Barke et al. 2018: IY | .53^***^ | .47^***^ | .67^***^ | - | - | - | .38^**^ |
| Thompson et al. 2009 | .53^***^ | .47^***^ | .65^***^ | - | - | .38 | .39^**^ |
| Van den Hoofdakker et al. 2007 | .54^***^ | .47^***^ | - | - | - | - | .40^**^ |

*Note*. Table shows effect sizes of meta-analysis when the referred study was left out (i.e. leave-one-out) to estimate the influence of single studies. As a comparison, the first row represents the overall effect size. *** *p* < .001, ** *p* < .01, * *p* < .05.

**Appendix S13: Sensitivity Analyses on Discontinuation Rates**

**Table S6.1.** *Sensitivity analyses to check for possible impact of study discontinuation rates*

|  |  | **All studies** | | | **High discontinuation rate group excluded** | | |
| --- | --- | --- | --- | --- | --- | --- | --- |
|  |  | ***k*** | ***n*-es** | **ES** | ***k*** | ***n*-es** | **ES** |
| **ADHD symptoms** | Within pre – follow-up | 23^+^ | 72^+^ | .53^***^ | 18^+^ | 52^+^ | .50^***^ |
|  | Within post – follow-up | 22 | 69 | .06 | 17 | 49 | .03 |
|  | Within pre – post | 22 | 69 | .53^***^ | 17 | 49 | .53^***^ |
| **Behavioural problems** | Within pre – follow-up | 26 | 52 | .47^***^ | 19 | 32 | .46^***^ |
|  | Within post – follow-up | 26 | 52 | .02 | 19 | 32 | .01 |
|  | Within pre – post | 26 | 52 | .47^***^ | 19 | 32 | .45^***^ |
| **Positive parenting** | Within pre – follow-up | 14 | 21 | .66^***^ | 10 | 15 | .77^**^ |
|  | Within post – follow-up | 14 | 21 | -.06 | 10 | 15 | -.05 |
|  | Within pre – post | 14 | 21 | .70^***^ | 10 | 15 | .81^***^ |
| **Negative parenting** | Within pre – follow-up | 9 | 20 | .79^***^ | 6 | 11 | .94^***^ |
|  | Within post – follow-up | 9 | 20 | .01 | 6 | 11 | .03 |
|  | Within pre – post | 9 | 20 | .79^***^ | 6 | 11 | .99^***^ |
| **Parenting sense of competence** | Within pre – follow-up | 13^+^ | 21^+^ | .54^***^ | 8^+^ | 14^+^ | .47^*^ |
|  | Within post – follow-up | 12 | 19 | -.03 | 7 | 12 | -.04 |
|  | Within pre – post | 12 | 19 | .68^***^ | 7 | 12 | .64^***^ |
| **Parent-child relationship quality** | Within pre – follow-up | 10 | 13 | .34^*^ | 8 | 11 | .18^*^ |
|  | Within post – follow-up | 10 | 13 | -.02 | 8 | 11 | -.09 |
|  | Within pre – post | 10 | 13 | .38^**^ | 8 | 11 | .31^*^ |
| **Parental mental health** | Within pre – follow-up | 14^+^ | 18^+^ | .39^**^ | 11^+^ | 13^+^ | .32^*^ |
|  | Within post – follow-up | 13 | 17 | -.01 | 10 | 12 | -.01 |
|  | Within pre – post | 13 | 17 | .45^***^ | 10 | 12 | .39^***^ |

*Note*: Intervention conditions were classified as “high discontinuation rate group” if 20% or more of the participants did not complete the later follow-up measurement. *k* = number of studies, *n-es* = number of effect sizes. Effect sizes (ES) represent standardized mean change scores using change score standardization with a correlation of *r* = .50. Positive effect sizes indicate beneficial effects of the intervention for all outcomes.

****p* < .001, ***p* < .01, **p* < .05.

^+^: Sonuga-Barke et al. (2004) only reported pre-measurement and later follow-up, and therefore the number of studies and effect sizes differs from the other comparisons.

**Appendix S14 – Predictor Analyses**

**Table S7.1.** *Results of meta-regression analyses on ADHD symptoms*

|  | **ADHD** | | | | | | | |
| --- | --- | --- | --- | --- | --- | --- | --- | --- |
|  | **PRE-FU** | | | | **POST-FU** | | | |
|  | *k* (*n*-*es*) | $\hat{\beta}$*_0_* | $\hat{\beta}$*_1_* | *I^2^* (2, 3) | *k* (*n*-*es*) | $\hat{\beta}$*_0_* | $\hat{\beta}$*_1_* | *I^2^* (2, 3) |
| **Participant characteristics** | |  |  |  |  |  |  |  |
| Age (con) | 21 (64) |  | -.01 | 47.8, 41.8 | 20 (61) |  | .01 | 50.4, 13.3 |
| Age (cat): Preschool | 12 (43) | .55^***^ |  | 50.4, 38.2 | 11 (40) | .06 |  | 45.4, 17.5 |
| Age (cat): School-age | 11 (29) | .51^***^ |  |  | 11 (29) | .07 |  |  |
| Age (cat): Preschool vs. School-age | 23 (72) |  | .04 |  | 22 (69) |  | -.01 |  |
| Sex (% boys) | 21 (66) |  | -.02^**^ | 68.3, 15.3 | 21 (66) |  | .00 | 47.1, 17.3 |
| Comorbid beh. dis. (%) | 17 (56) |  | -.00 | 49.8, 39.0 | 17 (56) |  | .00 | 53.5, 13.7 |
| Initial int. suc. ADHD | 22 (69) |  | .53^***^ | 79.6, 2.6 | 22 (69) |  | -.11 | 48.6, 12.3 |
| Initial int. suc. BP | 20 (65) |  | .44^*^ | 61.7, 23.2 | 20 (65) |  | -.16 | 49.4, 11.7 |
| Initial int. suc. PP | 10 (37) |  | .28 | 63.7, 23.9 | 10 (37) |  | -.03 | 63.3, 0.0 |
| Initial int. suc. NP | 4 (14) |  | -.33 | 42.8, 32.7 | 4 (14) |  | -.38 | 0.0, 17.8 |
| Initial int. suc. PSC | 7 (29) |  | .19 | 81.9, 3.8 | 7 (29) |  | -.20 | 62.4, 0.0 |
| Initial int. suc. PCR | 9 (36) |  | .33 | 84.9, 0.0 | 9 (36) |  | -.20 | 43.7, 22.8 |
| Initial int. suc. PMH | 11 (28) |  | 1.05^**^ | 72.0, 6.5 | 11 (28) |  | .05 | 44.0, 15.6 |
| **Intervention characteristics** | |  |  |  |  |  |  |  |
| Format: PT | 16 (50) | .51^***^ |  | 50.1, 38.5 | 15 (47) | .04 |  | 48.1, 13.8 |
| Format: MM | 7 (22) | .57^***^ |  |  | 7 (22) | .10 |  |  |
| Format: PT vs. MM | 23 (72) |  | -.05 |  | 22 (69) |  | -.06 |  |
| Duration | 23 (72) |  | .00 | 50.1, 38.5 | 22 (69) |  | -.00 | 45.3, 17.7 |
| **Study characteristics** |  |  |  |  |  |  |  |  |
| Control: Active | 13 (37) | .49^***^ |  | 51.0, 37.5 | 13 (37) | .11^**^ |  | 48.5, 11.8 |
| Control: Passive | 10 (35) | .58^***^ |  |  | 9 (32) | -.00 |  |  |
| Control: Active vs. Passive | 23 (72) |  | -.09 |  | 22 (69) |  | .11 |  |
| Study quality: % high | 23 (72) |  | .00 | 50.6, 38.0 | 22 (69) |  | .00 | 45.5, 17.2 |
| Study quality: % low | 23 (72) |  | -.00 | 49.9, 38.8 | 22 (69) |  | .00 | 46.5, 14.3 |
| Time lag to follow-up | 23 (72) |  | -.00 | 50.0, 38.7 | 22 (69) |  | .01 | 46.0, 16.4 |
| Masking: Masked | 9 (21) | .49^***^ |  | 53.3, 34.6 | 8 (20) | .18^**^ |  | 24.3, 39.3 |
| Masking: Unmasked | 21 (45) | .58^***^ |  |  | 20 (43) | .00 |  |  |
| Masking: Masked vs. Unmasked | 23 (66) |  | -.09 |  | 22 (63) |  | .18^**^ |  |

*Note*: *k* = number of studies; *n-es* = number of effect sizes; $\hat{\beta}$*_0_* denotes the SMCC (in terms of Hedges’ *g*) for the category referred to in the first column; $\hat{\beta}$*_1_* denotes the change in SMCC when the moderator increases with one; *I^2^* represents between-variables and between-studies heterogeneity (level 2 and 3 respectively).

*** *p* < .001, ** *p* < .01, * *p* < .05.

***Participant characteristics:*** con = continuous; cat = categorical; Preschool = children younger than 8 years old; School-age = children older than 8 years old; Comorbid beh. dis. = comorbid behavioural disorders; Initial int. suc. = initial intervention success from pre-intervention to post-intervention; BP = behavioural problems; PP = positive parenting; NP = negative parenting; PSC = parenting sense of competence; PCR = parent-child relationship quality; PMH = parental mental health.

***Intervention characteristics:*** PT = parent training; MM = multimodal.

***Study characteristics:*** % high = percentage high risk in risk of bias assessment; % low = percentage low risk in risk of bias assessment.

**Table S7.2.** *Results of meta-regression analyses with delivery method as predictor for
ADHD symptoms*

|  | **ADHD** | | | | | |
| --- | --- | --- | --- | --- | --- | --- |
|  | **PRE-FU** | | | **POST-FU** | | |
| *I^2^ (level2, 3)* | 55.5% | 31.8% |  | 45.1% | 18.3% |  |
|  | *k(n-es)* | $\hat{\beta}$*_0_* | $\hat{\beta}$*_1_* | *k(n-es)* | $\hat{\beta}$*_0_* | $\hat{\beta}$*_1_* |
| Group | 8 (19) | .34^**^ |  | 8 (19) | .03 |  |
| Individual | 11 (41) | .58^***^ |  | 10 (38) | .05 |  |
| Mixed | 4 (12) | .73^***^ |  | 4 (12) | .12 |  |
| Group vs. Individual |  | | -.24 |  | | -.02 |
| Group vs. Mixed |  |  | -.39^*^ |  |  | -.09 |
| Individual vs. Mixed |  |  | -.15 |  |  | -.07 |

*Note*: *k* = number of studies; *n-es* = number of effect sizes; $\hat{\beta}$*_0_* denotes the SMCC (in terms of Hedges’ *g*) for the category referred to in the first column; $\hat{\beta}$*_1_* denotes the change in SMCC when the moderator increases with one; *I^2^* represents between-variables and between-studies heterogeneity (level 2 and 3 respectively).

*** *p* < .001, ** *p* < .01, * *p* < .05.

**Table S7.3.** *Results of meta-regression analyses with rater type as predictor for ADHD symptoms*

|  | **ADHD** | | | | | |
| --- | --- | --- | --- | --- | --- | --- |
|  | **PRE-FU** | | | **POST-FU** | | |
| *I^2^* (level 2, 3) | 29.8% | 56.7% |  | 44.2% | 14.8% |  |
|  | *k(n-es)* | $\hat{\beta}$*_0_* | $\hat{\beta}$*_1_* | *k(n-es)* | $\hat{\beta}$*_0_* | $\hat{\beta}$*_1_* |
| Parent | 21 (39) | .62^***^ |  | 20 (37) | .02 |  |
| Teacher | 12 (17) | .45^***^ |  | 12 (17) | .14^**^ |  |
| Coder | 7 (9) | .04 |  | 7 (9) | .13 |  |
| Clinician | 5 (7) | .84^***^ |  | 4 (6) | -.02 |  |
| Parent vs. Teacher |  |  | .17^*^ |  |  | -.11^*^ |
| Parent vs. Coder |  |  | .58^***^ |  |  | -.11 |
| Parent vs. Clinician |  |  | -.22 |  |  | .04 |
| Teacher vs. Coder |  |  | .42^**^ |  |  | .00 |
| Teacher vs. Clinician |  |  | -.39^**^ |  |  | .15 |
| Coder vs. Clinician |  |  | -.80^***^ |  |  | .15 |

*Note*: *k* = number of studies; *n-es* = number of effect sizes; $\hat{\beta}$*_0_* denotes the SMCC (in terms of Hedges’ *g*) for the category referred to in the first column; $\hat{\beta}$*_1_* denotes the change in SMCC when the moderator increases with one; *I^2^* represents between-variables and between-studies heterogeneity (level 2 and 3 respectively).

*** *p* < .001, ** *p* < .01, * *p* < .05.

**Table S7.4.** *Results of meta-regression analyses on behavioural problems*

|  | **Behavioural problems** | | | | | | | |
| --- | --- | --- | --- | --- | --- | --- | --- | --- |
|  | **PRE-FU** | | | | **POST-FU** | | | |
|  | *k* (*n*-*es*) | $\hat{\beta}$*_0_* | $\hat{\beta}$*_1_* | *I^2^* (2, 3) | *k* (*n*-*es*) | $\hat{\beta}$*_0_* | $\hat{\beta}$*_1_* | *I^2^* (2, 3) |
| **Participant characteristics** | |  |  |  |  |  |  |  |
| Age (con) | 24 (48) |  | -.05^**^ | 4.8, 78.9 | 24 (48) |  | -.01 | 43.6, 25.7 |
| Age (cat): Preschool | 13 (32) | .58^***^ |  | 6.5, 77.5 | 13 (32) | .03 |  | 38.7, 29.0 |
| Age (cat): School-age | 13 (20) | .36^***^ |  |  | 13 (20) | -.00 |  |  |
| Age (cat): Preschool vs. School-age | 26 (52) |  | .22 |  | 26 (52) |  | .03 |  |
| Sex (% boys) | 25 (48) |  | -.00 | 13.2, 60.8 | 25 (48) |  | -.00 | 40.9, 28.6 |
| Comorbid beh. dis. (%) | 18 (38) |  | .00 | 4.8, 83.8 | 18 (38) |  | -.00 | 41.8, 30.0 |
| Initial int. suc. ADHD | 20 (44) |  | .59^***^ | 12.3, 62.1 | 20 (44) |  | -.02 | 48.8, 6.3 |
| Initial int. suc. BP | 26 (52) |  | .61^***^ | 11.7, 64.2 | 26 (52) |  | -.20 | 39.9, 24.1 |
| Initial int. suc. PP | 12 (31) |  | .35 | 3.1, 85.3 | 12 (31) |  | .06 | 52.8, 0.0 |
| Initial int. suc. NP | 7 (11) |  | .29 | 24.7, 28.1 | 7 (11) |  | -.09 | 0.0, 0.0 |
| Initial int. suc. PSC | 9 (21) |  | .31 | 23.1, 26.0 | 9 (21) |  | -.11 | 42.1, 2.6 |
| Initial int. suc. PCR | 10 (20) |  | .41 | 22.6, 42.0 | 10 (20) |  | -.05 | 33.8, 44.8 |
| Initial int. suc. PMH | 12 (27) |  | .87 | 4.3, 83.4 | 12 (27) |  | -.14 | 30.9, 0.0 |
| **Intervention characteristics** | |  |  |  |  |  |  |  |
| Format: PT | 20 (43) | .56^***^ |  | 7.5, 73.1 | 20 (43) | .04 |  | 38.2, 28.1 |
| Format: MM | 6 (9) | .17 |  |  | 6 (9) | -.09 |  |  |
| Format: PT vs. MM | 26 (52) |  | .40^**^ |  | 26 (52) |  | .13 |  |
| Duration | 26 (52) |  | -.00 | 5.9, 79.9 | 26 (52) |  | -.00 | 33.5, 34.5 |
| **Study characteristics** |  |  |  |  |  |  |  |  |
| Control: Active | 14 (25) | .35^***^ |  | 7.4, 75.5 | 14 (25) | .05 |  | 32.1, 36.0 |
| Control: Passive | 12 (27) | .63^***^ |  |  | 12 (27) | -.04 |  |  |
| Control: Active vs. Passive | 26 (52) |  | -.28^*^ |  | 26 (52) |  | .10 |  |
| Study quality: % high | 26 (52) |  | -.00 | 5.9, 79.9 | 26 (52) |  | -.00 | 38.3, 29.2 |
| Study quality: % low | 26 (52) |  | -.00 | 6.0, 79.5 | 26 (52) |  | .00 | 40.1, 24.9 |
| Time lag to follow-up | 26 (52) |  | -.03 | 6.2, 79.1 | 26 (52) |  | .02 | 37.8, 28.7 |
| Masking: Masked | 9 (11) | .49^***^ |  | 6.9, 77.1 | 9 (11) | .17^**^ |  | 48.5, 6.6 |
| Masking: Unmasked | 17 (38) | .48^***^ |  |  | 17 (38) | -.04 |  |  |
| Masking: Masked vs. Unmasked | 26 (49) |  | .01 |  | 26 (49) |  | .21^**^ |  |

*Note*: *k* = number of studies; *n-es* = number of effect sizes; $\hat{\beta}$*_0_* denotes the SMCC (in terms of Hedges’ *g*) for the category referred to in the first column; $\hat{\beta}$*_1_* denotes the change in SMCC when the moderator increases with one; *I^2^* represents between-variables and between-studies heterogeneity (level 2 and 3 respectively).

*** *p* < .001, ** *p* < .01, * *p* < .05.

***Participant characteristics:*** con = continuous; cat = categorical; Preschool = children younger than 8 years old; School-age = children older than 8 years old; Comorbid beh. dis. = comorbid behavioural disorders; Initial int. suc. = initial intervention success from pre-intervention to post-intervention; BP = behavioural problems; PP = positive parenting; NP = negative parenting; PSC = parenting sense of competence; PCR = parent-child relationship quality; PMH = parental mental health.

***Intervention characteristics:*** PT = parent training; MM = multimodal.

***Study characteristics:*** % high = percentage high risk in risk of bias assessment; % low = percentage low risk in risk of bias assessment.

**Table S7.5.** *Results of meta-regression analyses with delivery method as predictor for behavioural problems*

|  | **Behavioural problems** | | | | | |
| --- | --- | --- | --- | --- | --- | --- |
|  | **PRE-FU** | | | **POST-FU** | | |
| *I^2^* (level 2, 3) | 5.7% | 80.6% |  | 35.1% | 33.6% |  |
|  | *k(n-es)* | $\hat{\beta}$*_0_* | $\hat{\beta}$*_1_* | *k(n-es)* | $\hat{\beta}$*_0_* | $\hat{\beta}$*_1_* |
| Group | 11 (18) | .48^***^ |  | 11 (18) | .04 |  |
| Individual | 11 (27) | .50^***^ |  | 11 (27) | -.01 |  |
| Mixed | 4 (7) | .38 |  | 4 (7) | .03 |  |
| Group vs. Individual |  |  | -.02 |  |  | .06 |
| Group vs. Mixed |  |  | .10 |  |  | .01 |
| Individual vs. Mixed |  |  | .12 |  |  | -.04 |

*Note*: *k* = number of studies; *n-es* = number of effect sizes; $\hat{\beta}$*_0_* denotes the SMCC (in terms of Hedges’ *g*) for the category referred to in the first column; $\hat{\beta}$*_1_* denotes the change in SMCC when the moderator increases with one; *I^2^* represents between-variables and between-studies heterogeneity (level 2 and 3 respectively).

*** *p* < .001, ** *p* < .01, * *p* < .05.

**Table S7.6.** *Results of meta-regression analyses with rater type as predictor for behavioural problems*

|  | **Behavioural problems** | | | | | |
| --- | --- | --- | --- | --- | --- | --- |
|  | **PRE-FU** | | | **POST-FU** | | |
| *I^2^* (level 2, 3) | 2.5% | 81.8% |  | 39.3% | 9.1% |  |
|  | *k(n-es)* | $\hat{\beta}$*_0_* | $\hat{\beta}$*_1_* | *k(n-es)* | $\hat{\beta}$*_0_* | $\hat{\beta}$*_1_* |
| Parent | 21 (35) | .47^***^ |  | 21 (35) | -.05 |  |
| Teacher | 8 (11) | .35^***^ |  | 8 (11) | .11 |  |
| Coder | N/A | N/A |  | N/A | N/A |  |
| Clinician | N/A | N/A |  | N/A | N/A |  |
| Parent vs. Teacher |  |  | .12^*^ |  |  | -.16^*^ |

*Note*: *k* = number of studies; *n-es* = number of effect sizes; $\hat{\beta}$*_0_* denotes the SMCC (in terms of Hedges’ *g*) for the category referred to in the first column; $\hat{\beta}$*_1_* denotes the change in SMCC when the moderator increases with one; *I^2^* represents between-variables and between-studies heterogeneity (level 2 and 3 respectively).

*** *p* < .001, ** *p* < .01, * *p* < .05.

**Appendix S15 – Sensitivity Analyses: Masking**

**Table S8.1.** *Sensitivity analyses masking as predictor on within-group effect sizes from post-intervention to follow-up*

|  | **ADHD  POST-FU** | | | | **Behavioural Problems  POST-FU** | | | |
| --- | --- | --- | --- | --- | --- | --- | --- | --- |
|  | *k* (*n*-*es*) | $\hat{\beta}$*_0_* | $\hat{\beta}$*_1_* | *I^2^* (2, 3) | *k* (*n*-*es*) | $\hat{\beta}$*_0_* | $\hat{\beta}$*_1_* | *I^2^* (2, 3) |
| Masking: Masked | 6 (15) | .17^**^ |  | 65.3, 0.0 | 4 (6) | .10 |  | 60.6, 0.0 |
| Masking: Unmasked | 6 (8) | -.09 |  |  | 4 (6) | .03 |  |  |
| Masking: Masked vs. Unmasked | 6 (23) |  | .26^**^ |  | 4 (12) |  | .08 |  |

*Note*: *k* = number of studies; *n-es* = number of effect sizes; $\hat{\beta}$*_0_* denotes the SMCC (in terms of Hedges’ *g*) for the category referred to in the first column; $\hat{\beta}$*_1_* denotes the change in SMCC when the moderator increases with one; *I^2^* represents between-variables and between-studies heterogeneity (level 2 and 3 respectively).

*** *p* < .001, ** *p* < .01, * *p* < .05.
